# Is health research undertaken where the burden of disease is greatest? Observational study of geographical inequalities in recruitment to research in England 2013–2018

**DOI:** 10.1186/s12916-020-01555-4

**Published:** 2020-05-18

**Authors:** Peter Bower, Christos Grigoroglou, Laura Anselmi, Evangelos Kontopantelis, Matthew Sutton, Mark Ashworth, Philip Evans, Stephen Lock, Stephen Smye, Kathryn Abel

**Affiliations:** 1 NIHR Clinical Research Network, University of Manchester, Manchester, UK; 2 NIHR School for Primary Care Research, University of Manchester, Manchester, UK; 3 School of Population Health and Environmental Sciences, King’s College London, Manchester, UK; 4 General Practice and Primary Care, College of Medicine and Health, University of Exeter, Manchester, UK; 5 NIHR CRN National Specialty Lead for Primary Care and Cluster C Lead, Kings College, London, UK; 6 NIHR Clinical Research Network Business Intelligence, Manchester, UK; 7 NIHR CRN Specialty Cluster Lead, CRN National Coordinating Centre (CRNCC), NIHR Clinical Research Network (CRN), Kings College London, London, UK

**Keywords:** Research activity, Recruitment, Equity

## Abstract

**Background:**

Research is fundamental to high-quality care, but concerns have been raised about whether health research is conducted in the populations most affected by high disease prevalence. Geographical distribution of research activity is important for many reasons. Recruitment is a major barrier to research delivery, and undertaking recruitment in areas of high prevalence could be more efficient. Regional variability exists in risk factors and outcomes, so research done in healthier populations may not generalise. Much applied health research evaluates interventions, and their impact may vary by context (including geography). Finally, fairness dictates that publically funded research should be accessible to all, so that benefits of participating can be fairly distributed. We explored whether recruitment of patients to health research is aligned with disease prevalence in England.

**Methods:**

We measured disease prevalence using the Quality and Outcomes Framework in England (total long-term conditions, mental health and diabetes). We measured research activity using data from the NIHR Clinical Research Network. We presented descriptive data on geographical variation in recruitment rates. We explored associations between the recruitment rate and disease prevalence rate. We calculated the share of patient recruitment that would need to be redistributed to align recruitment with prevalence. We assessed whether associations between recruitment rate and disease prevalence varied between conditions, and over time.

**Results:**

There was significant geographical variation in recruitment rates. When areas were ranked by disease prevalence, recruitment was not aligned with prevalence, with disproportionately low recruitment in areas with higher prevalence of total long-term and mental health conditions. At the level of 15 local networks, analyses suggested that around 12% of current recruitment activity would need to be redistributed to align with disease prevalence. Overall, alignment showed little change over time, but there was variation in the trends over time in individual conditions.

**Conclusions:**

Geographical variations in recruitment do not reflect the suitability of the population for research. Indicators should be developed to assess the fit between research and need, and to allow assessment of interventions among funders, researchers and patients to encourage closer alignment between research activity and burden.

**Electronic supplementary material:**

The online version of this article (10.1186/s12916-020-01555-4) contains supplementary material, which is available to authorized users.

## Background

Research is fundamental to high-quality health care, providing the evidence-base for health policy and treatment delivery. In addition, taking part in research is itself associated with better performance among health care organisations [1, 2], as well as being a positive experience for patients [3].

Nevertheless, delivering research is challenging, with various factors impacting on patient recruitment and retention [4]. Difficulties with recruitment may be greater in certain contexts, such as deprived settings [5] and among ethnic minorities [6]. Equally, barriers may reflect the characteristics of organisations, in terms of their expertise and research infrastructure. Organisations may focus on populations that are easier to recruit, ignoring populations that are considered ‘hard to reach’ [7]. These barriers and perceptions mean that the patients recruited into health research may not accurately reflect the populations to which the results are expected to apply [8, 9], leading to concerns about ‘research waste’ [10].

England is unique in having a national research infrastructure (the NIHR Clinical Research Network) that provides national coverage in opportunities for research participation [11, 12]. The network comprises 15 Local Clinical Research Networks (LCRNs) covering England. This network has been responsible for recruitment of several million patients since its inception. This network provides support to interventional and observational studies across 30 specialties, meeting the costs of staff, facilities, equipment and support services. Studies eligible for support include medical studies, health services research, public health and social care.

Although the total number of recruits represents a key measure of the performance of a research network, it is equally critical to ensure that the patients recruited are representative of the wider population [13]. Although the network has national coverage in principle, barriers to recruitment mean that primary research (involving patients consenting to take part) rarely approaches the national coverage possible with routine administrative data. This has led to concerns about the degree to which health research is ‘conducted with and in the populations most affected’ [14], i.e. that the geographical distribution of research activity does not reflect the underlying prevalence of conditions in the population.

The prevalence of a disorder is a key driver of the impact of disease on local populations. Demographic shifts mean that disorders are increasingly prevalent among older populations [15] and such populations are increasingly found in rural and coastal areas [16]. In contrast, research may be clustered around locations associated with research units, major hospitals and universities.

Conventionally, the geographical distribution of research activity has not been seen as critical to recruitment of a representative sample of patients. As long as the characteristics of the recruited sample reflect the underlying population, in principle it should not matter where those patients have been recruited. Although true in principle, there is good evidence that recruited patients do not generally reflect the underlying population [8, 9, 17, 18]. We argue that geographical distribution of recruitment may be important for a number of reasons.

First, recruitment is a major barrier to delivery of research [19]. All other things being equal, undertaking recruitment in areas of high prevalence could be more efficient, as there will be more people to screen for eligibility.

Secondly, regional variability exists in the UK in risk factors and outcomes, with the North-South divide in premature mortality being a prime example [20]. Two thirds of this divide have been attributed to measured deprivation, but one third is likely due to other factors: cultural differences, health-related behaviour, or social fragmentation [21]. Research done in healthier populations may not generalise to populations facing greater challenges to health and well-being.

Thirdly, a significant proportion of applied health research is concerned with the evaluation of interventions, and the impact of interventions may vary according to context [22–24].

Finally, fairness dictates that publically funded research should be accessible to all. Only in this way can the additional benefits of participating in research (such as improved quality of care and patient outcomes) be made accessible to all [1, 2].

A strength of the research infrastructure in England is its focus on performance measurement, as the numbers of patients recruited is a core metric, and accurate recruitment data are available nationally over time. We used these data to explore the following research questions:

1. Is patient recruitment aligned with disease prevalence in England? How does the alignment vary across clinical specialities?
2. Is alignment getting better or worse over time?

## Methods

### Measures of recruitment

We analysed data on research that took place between 2013 and 2018 in England, obtained from NIHR Clinical Research Network Coordinating Centre (CRNCC) Business Intelligence for recruitment to NIHR portfolio studies in England, which includes trials and observational studies. Studies are assigned to clinical specialties (https://www.nihr.ac.uk/nihr-in-your-area/specialties.htm). Ethical approval was not needed for this secondary analysis.

We examined data at two levels: the 15 LCRNs which are the primary administrative units of the network, and the 195 clinical commissioning groups (CCGs) in England in 2018 (NHS organisations which plan services for particular geographies). CCGs can be mapped into the smaller number of LCRNs.

Patients recruited to studies on the network are assigned to one of the 15 regional LCRNs. Ideally, recruitment data would be based on the place of residence of each recruit, which would be matched to prevalence data on a similar basis. However, location of patient recruitment is recorded, not their home address, so the mapping of patients to units of analysis is not exact. Around 80% of recruitment is associated with a hospital Trust and can be reliably matched to LCRNs only. Around 20% of the recruitment is in primary care settings and can be reliably matched both to LCRNs and CCGs. We conducted an approximate mapping of hospital Trust recruitment data to CCGs, based on the location of patient recruitment, but this mapping will tend to attribute recruitment to CCGs hosting major research and tertiary centres. We therefore had three sets of data with different strengths and weaknesses (see Table 1). We present analysis of each data set and consider the consistency of results.

**Table 1:** Data types and their strengths and weaknesses

**Data type**	**Scope**	**Accuracy**	**Granularity**
Data on recruitment by hospital Trusts and primary care aggregated into CCGs	Comprehensive	Approximate	Higher
	All CRN recruitment data included	Recruitment from hospital trusts attributed to CCG based on location	Data available across 195 CCGs
Hospital trust and primary care recruitment data matched to LCRNs	Comprehensive	Accurate	Lower
	All CRN recruitment data included		Data aggregated to 15 LCRNs
Data on recruitment by primary care aggregated into CCGs	Partial	Accurate	Higher
	All primary care data, but around 20% of CRN recruitment data included		Data available across 195 CCGs

### Measures of disease prevalence

Our measure of disease prevalence was data from the Quality and Outcomes Framework (QOF) disease registers in general practice [25] from 2013/14 to 2017/18. These registers are comprehensive and of acceptable quality [26]. QOF is the largest national primary care incentive scheme worldwide and even though participation is voluntary, around 95% of practices participate [25].

We examined data for all long-term conditions combined and separately for mental health and diabetes. The analysis of all conditions provides a broad overview of the relationship between disease prevalence and recruitment. Examining specific conditions allows us to explore whether those relationships vary in different diseases. Diabetes and mental health are both high-prevalence conditions important to health policy. Mental health research funding is known to be poorly matched to burden [27, 28].

For analysis of all long-term conditions, we used data on patients with the following conditions: atrial fibrillation, asthma, cancer, coronary heart disease, chronic kidney disease, chronic obstructive pulmonary disease, dementia, depression, diabetes, epilepsy, heart failure, hypertension, limiting disabilities, severe mental health, obesity, osteoporosis, peripheral arterial disease, rheumatoid arthritis, and stroke. For mental health, we combined data on common mental disorders (depression) and severe mental disorders (psychosis, schizophrenia and bipolar disorder). For each unit, we calculated prevalence by using the sum on the relevant disease registers over the total population, for each relevant time period. Multimorbidity is common, especially among older patients [15]. We used an aggregate measure of morbidity, adding separate condition prevalence estimates (assuming, therefore, that the effects of multiple conditions are additive and that each condition is equally important in terms of overall disease prevalence).

We measured disease prevalence at the LCRN and CCG levels. QOF data were available at the practice level and were attributed to both the CCG and LCRN levels. QOF data are provided each year and allow for CCG mergers that occurred throughout our study period. However, the mapping of study recruits from secondary care was based on 2018 CCG areas (*n* = 195). For this reason, QOF data prior to 2018 were aggregated into the larger 2018 merged CCGs for the whole study period. Data on disease burden were complete for all CCGs in each year. All QOF data were obtained from NHS Digital for the study period 2013–2018 [25].

### Data analysis

*Is patient recruitment aligned with disease prevalence in England? How does the alignment vary across clinical specialities?*

We conducted this analysis in two phases. First, we assessed whether there was substantial geographical variation in the recruitment rate across CCGs and LCRNs.

We calculated the recruitment rate as the number of recruits divided by the number of people within each disease group (all long-term conditions, mental health and diabetes). We produced maps to illustrate the geographical distribution of recruitment.

**Fig. 1:**
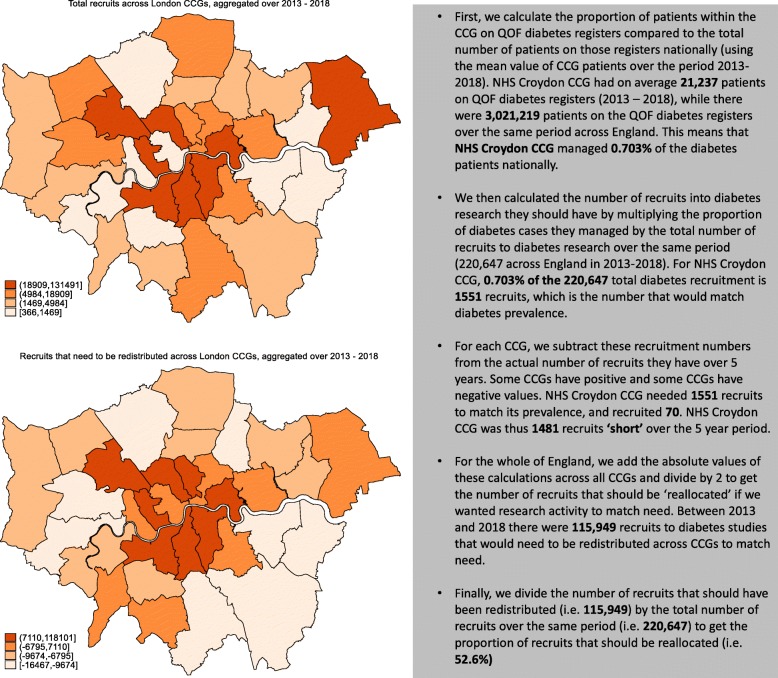
Calculation of the redistribution index

We calculated a ‘redistribution index’ to quantify the share of total recruits that would have to be redistributed between over-recruiting to under-recruiting areas to achieve equal shares of recruitment and disease prevalence. To assess redistribution, we used the absolute value of difference between the actual and the equitable number of recruits across each area (see Fig. 1 for details of the calculations).

In the second set of analyses, we explored whether there was a systematic association between the recruitment rate and the disease prevalence rate. We calculated the prevalence rate as the number of people with the condition divided by the population of the area (when we are combining more than one health condition, this measure can exceed one in areas with high levels of disease). We divided the 195 CCGs into quintiles of the prevalence rate to simplify presentation. We ranked units (CCG quintiles or individual LCRNs) from low to high based on the prevalence rate. We used bar charts for CCGs and LCRNs, to show whether the recruitment rate varied at different levels of the prevalence rate.

We present separate analyses of all long-term conditions, mental health and diabetes, to explore whether the patterns found across conditions were replicated in individual conditions.

*Is alignment getting better or worse over time?*

To capture change over time, we used concentration curves [29]. A concentration curve plots the cumulative percentage of one variable against the cumulative percentage of the sample, ranked by another variable. Concentration curves are used to represent the extent of inequity, captured by divergence from the 45 degree line (line of equity).

We plotted the cumulative recruitment rate within each of our disease groups against the cumulative proportion of our geographical units (CCGs or LCRNs), ranked from the lowest to highest prevalence. If recruitment rate is constant across geographical units with different prevalence rates, the curve will be at the line of equality [30]. The curve lies above the 45° line, when there is a disproportionate concentration of recruitment in lower prevalence areas, and below the line when the opposite is true.

We plotted concentration curves and calculated the related redistribution indices for our three groups of conditions for each year (2013/14 to 2017/18) to provide a visual assessment of change over time. We also ran a time series regression with the redistribution index for each disease group as the dependent variable and time as the independent variable and to test whether the linear trend in the redistribution index was statistically different from zero.

## Results

We analysed our three groups of conditions (all long-term conditions, mental health and diabetes) using the three data types identified in Table 1.

*Is recruitment aligned with disease prevalence in England? How does the alignment vary across clinical specialities?*

Table 2 shows the recruitment rate per 1000 people with the condition for our three disease groups. There was significant variation in recruitment rates across different geographical areas. For example, using all data and all conditions, 25% of CCGs reported only a quarter of the mean recruitment rate across England (5.74 per 1000 compared to 19.56 per 1000) and 10% of CCGs had nearly double the mean rate. Similar relationships were found when using data on primary care recruitment only (for example, 25% of CCGs had rates of 1.19 or less per 1000, compared to a mean of 3.22). This variation was also found to an even greater degree in relation to mental health and diabetes. For example, using all data, 25% of CCGs had a mean rate of recruitment in mental health of 0.44 or lower (compared to a mean of 8.26), while in diabetes 25% of CCGs had a mean rate of 1.74 (compared to a mean of 14.61).

**Table 2:** Descriptive statistics on recruitment

	**Primary care and trust data, CCG level**	**Primary care and trust data, LCRN level**	**Primary care data, CCG level**
Recruitment rate* **Mean (SD), interquartile range (IQR) [25% and 75% centiles]** **across CCGs/LCRNs**			
All conditions	19.56, [5.74, 23.52]	19.56, [16.25, 22.67]	3.22, [1.19, 3.59]
Mental health	8.26, [0.44, 13.83]	8.26, [6.31, 10.88]	0.77, [0.06, 0.68]
Diabetes	14.61, [1.74, 15.12]	14.61, [10.82, 21.95]	2.65, [0.18, 3.21]
**Recruitment redistribution index**^**¥**^ **% (Redistributed recruits/total recruits) × 100**			
All conditions	43.3% (1,416,436/3,272,538)	12.0% (394,290/3,272,538)	36.2% (194,389/539,046)
Mental health	53.3% (117,133/219,966)	12.6% (27,856/219,966)	58.9% (12,091/20,538)
Diabetes	52.6% (115,949/220,647)	23.8% (52,473/220,647)	57.1% (22,838/40,030)

^*^Recruitment rate is calculated as the number of recruits divided by the number of people within each disease group

^¥^Redistribution index is the sum across areas of the absolute value of the difference between the actual and the equitable number of recruits, divided by two

Table 2 also shows the numbers of recruits who would need to be reallocated across CCGs and LCRNs, based on our three groups of conditions and data types. In relation to all conditions at the level of the CCG, the percentage was 43%, compared to 53% for mental health and diabetes. The redistribution percentages were much lower for the larger LCRN areas (12–24%).

**Fig. 2:**
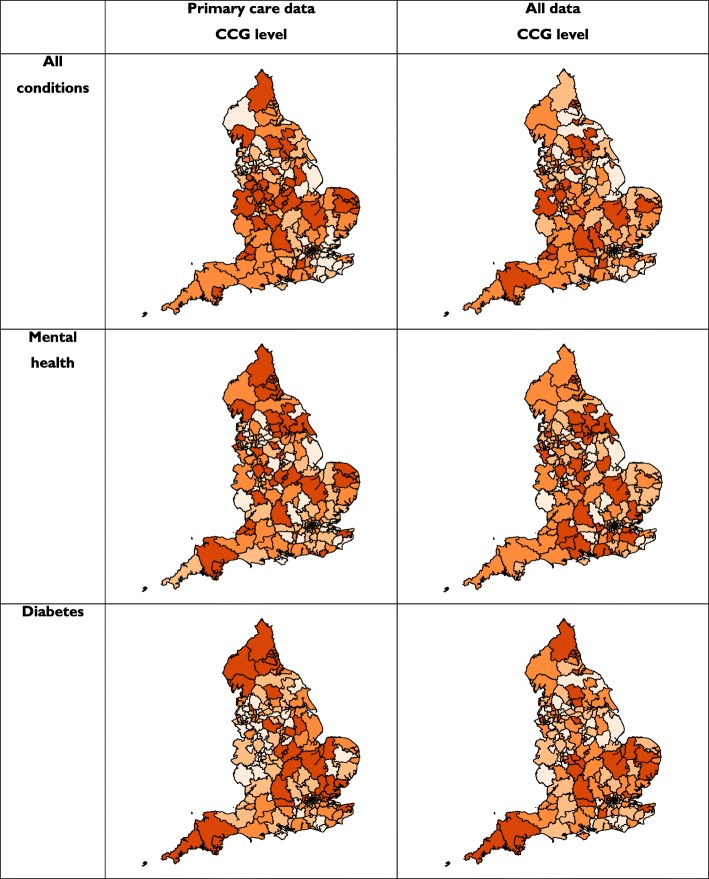
Geographical distribution of recruitment per 1000 people across 195 CCGs

Figure 2 shows the geographical distribution of recruitment per 1000 people with the condition across the 195 CCGs in England (detailed figures are in Additional file 1, Appendices 1a to 1f).

**Fig. 3:**
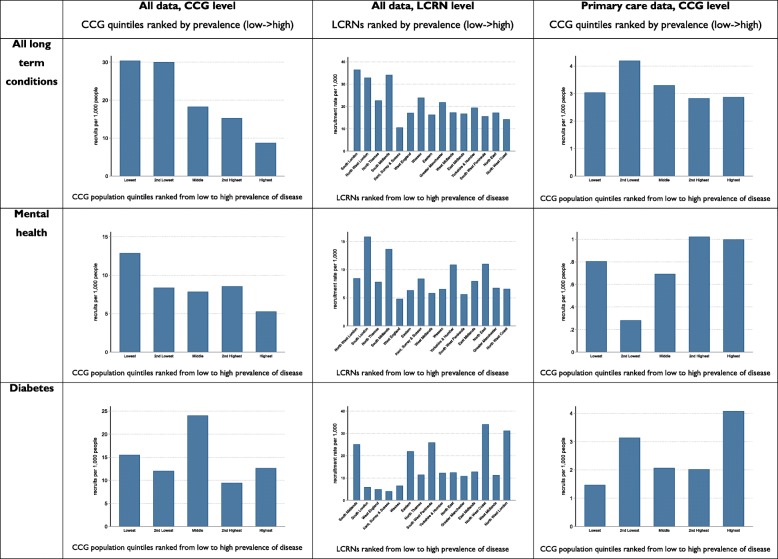
Bar charts for CCGs or LCRNs ranked by prevalence of disease

Figure 3 displays bar charts for all 9 analyses, with units of analysis (CCG or LCRN) ranked by prevalence of disease (detailed figures are in the Additional file 1 Appendices 2a to 2i). If the recruitment rate was aligned with the prevalence rate, recruitment rates (plotted in the *Y* axis) would remain constant as the underlying prevalence increases (along the *X* axis).

In the first row of Fig. 3, data on all long-term conditions showed broadly similar patterns, with higher rates of recruitment in areas of lower underlying prevalence in both CCG and LCRN analyses using all recruitment data. When CCGs were analysed using primary care data only, the patterns were slightly more complex, with broadly similar recruitment across quintiles of prevalence, with the exception in the second lowest quintile.

In the second row of Fig. 3, data on mental health showed a similar pattern of higher rates of recruitment in lower prevalence areas at the CCG level. The patterns were more mixed at the LCRN level. When the analysis was restricted to primary care data alone, there was a broad pattern of increasing rates of recruitment in areas of higher prevalence, although there was evidence of misalignment in the lowest quintile of prevalence.

In the third row of Fig. 3, data on diabetes show a different pattern. The highest levels of recruitment are among those CCGs with moderate prevalence, while high rates of recruitment were found in LCRNs with low, moderate and high prevalence.

**Fig. 4:**
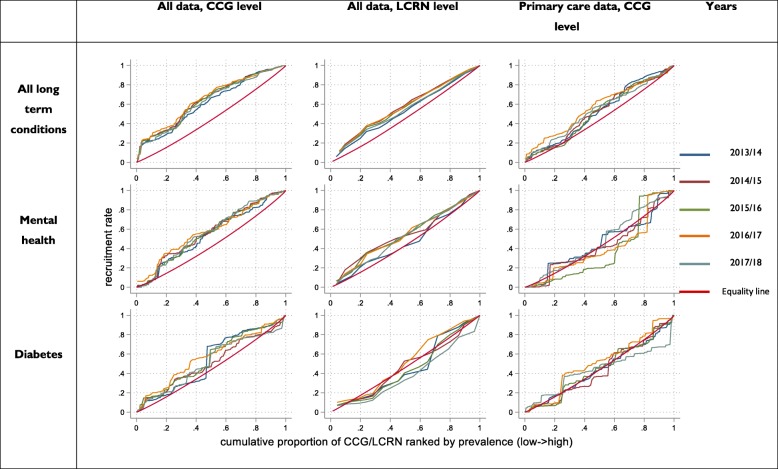
Concentration curves for recruitment

Figure 4 shows the concentration curves for all long-term conditions over a 5-year period (detailed figures are in the Additional file 1 Appendices 3a to 3i). Using all recruitment data and all long-term conditions, the curves demonstrate a disproportionate concentration of recruitment in lower prevalence areas which is consistent over time. The curves for mental health and diabetes are more variable but reflect the same trends.

Table 3 shows the redistribution indices over time for each of the three condition groups. Redistribution indices for all conditions show only small changes over time. The redistribution indices for diabetes and mental health are more variable. The regression analysis of trends with time as the independent variable shows a more marked decrease in mental health in three of four datasets, while the opposite pattern is evident in diabetes.

**Table 3:** Trends in the redistribution of recruits over time, 2013–2018

**Year**	**CCG (primary care and Trust data)**	**CCG (primary care data only)**	**LCRN (primary care and Trust data)**	**LCRN (primary care data only)**
**All conditions**				
2013–2014	46%	39%	9%	18%
2014–2015	44%	44%	15%	19%
2015–2016	44%	45%	13%	19%
2016–2017	45%	42%	14%	18%
2017–2018	43%	42%	11%	24%
Trend†	−0.60	0.41	0.25	1.00
**Mental health**				
2013–2014	56%	75%	22%	54%
2014–2015	59%	72%	21%	46%
2015–2016	57%	72%	13%	50%
2016–2017	59%	69%	16%	40%
2017–2018	56%	53%	13%	31%
Trend†	0.04	−4.86	−2.22	−5.08
**Diabetes**				
2013–2014	56%	58%	36%	28%
2014–2015	55%	67%	25%	47%
2015–2016	59%	72%	26%	52%
2016–2017	57%	74%	26%	52%
2017–2018	60%	74%	27%	62%
Trend†	1.08	4.04	−1.65	7.13

† Trend parameter represents the linear trend in the redistribution index. All trends are statistically significant (full data are presented in Additional file 1, Appendix 4). Patients can be redistributed from over-recruiting areas to under-recruiting areas and vice versa according to the direction and magnitude of effects. A positive trend indicates that the percentage of recruits needing re-distribution is increasing (i.e. that alignment between recruitment and prevalence is worse)

## Discussion

### Statement of principal findings

Between 2013 and 2018, rates of recruitment to health research varied substantially across England. Across all long-term conditions, and specifically, in mental health and diabetes, there was evidence of a lack of alignment between recruitment and disease prevalence, with evidence of high rates of recruitment in some CCGs and LCRNs with low prevalence of disease and lower rates of recruitment in regions with the highest prevalence of disease. However, the patterns of recruitment are complex—areas with the lowest prevalence are not consistently the highest recruiting over the 5-year time period. The existence of substantial geographical variation in recruitment which is not related to prevalence of disease suggests that a key driver may be regional research capability rather than the suitability of the population for research. This may have implications for the quality of the research, and for the equitable distribution of the benefits of participation [1, 2, 31].

### Strengths and weaknesses of the study

Although access to detailed recruitment and prevalence data at a national level is unique, the data have limitations. Our analysis is focussed on applied health research, and the NIHR portfolio does not capture all applied health research. Studies on the portfolio are assigned to clinical specialities based on the study population and focus, but there may be studies which relate to diabetes and mental health which are not coded as such, and thus analysis by disease may be more vulnerable to such misclassification. Applied health research which does not consent participants (e.g., administrative datasets) is not captured.

As noted, mapping of most of the CRN recruitment data to CCGs is approximate, as recruitment location is recorded. Mapping recruitment to LCRN rather than CCG is more reliable, but less granular, as LCRN areas are large and estimates of the alignment between prevalence and recruitment at such a level may hide important relationships within areas. Although primary care recruitment data can be mapped more accurately to individual CCGs, this represents only around 20% of recruitment. We have assessed the consistency across these sources. The fact that primary care data mapped to CCG shows somewhat different patterns to the total data suggests some caution in interpretation. Finally, the spatial maps are not weighted by locality size, and hence, rural areas dominate the maps. This makes it easier to identify the areas, but takes no account of population density.

Data on disease reflects prevalence of long-term conditions captured in general practice systems only, focussed on long-term conditions which account for much health care activity [32, 33]. This allowed us to use more precise, timely and comprehensive information for determining prevalence at a local level. However, we have assessed prevalence, not burden of disease, associated disability or the distribution of risk factors [34, 35]. Measurement of prevalence through the QOF has limitations. First, QOF includes data only for certain chronic conditions and our measure of need does not fully capture overall burden. Second, we were unable to measure multimorbidity and used an aggregate measure of disease burden assuming that burden is additive. Multimorbidity may be associated with higher or lower impact depending on the conditions involved [36, 37]. There is no strong consensus on the optimal ways to describe patterns of multimorbidity. Although our method of dealing with multimorbidity would influence estimates of disease prevalence, it would be less likely to affect our assessment of the links between prevalence and recruitment unless patterns of multimorbidity varied by geography. Our data on disease burden does not capture incidence which may be more important in certain clinical areas. The data may also be subject to the effects of associated financial incentives which may impact on case finding. Despite these limitations, QOF data are the only source of information of prevalence of disease on a national and comprehensive basis. Future analyses could explore alternative measures of disease prevalence, such as per capita, measures of deprivation, and by synthetic measures of burden. Collection of data on recruited patient’s place of residence would also allow more accurate analysis of the link between research activity and burden.

### Meaning of the study: possible mechanisms and implications for clinicians or policymakers

The stimulus for this research was the proposal that research delivery was poorly aligned with need [14]. This has been our focus, although it should be noted that research in general and clinical research in particular could be less than optimally distributed across patient populations, among disease severity within the patient population, across clinical providers and across research organisations. These distinctions are important because the impact of each (and the ways to remedy this) may differ. Interpretation is also dependent on a number of issues. These may include the reliability of our prevalence measures by region as well as over time, alongside value judgments about what recruitment activity should look like across a service, region or population.

Although we have assumed that recruitment should reflect underlying prevalence of disease within a region, this is complicated by the organisation of services within the NHS. People with particular conditions (e.g. specific cancers) may travel out of region to specialist research centres. Such centres may be located in lower prevalence regions for that condition while serving bigger, possibly more severe members of a disease population. The importance of the geographical distribution of research may vary by the type of research and may be less of an issue for surgical interventions and short-term outcomes, where potential confounders like socio-economic deprivation may have less impact.

There are a variety of other issues to take account of. The NIHR Clinical Research Network has an underlying philosophy of equality of access to research opportunities and there may be tensions between that mission and measures to re-align recruitment activity with population prevalence. Equally, there may be an argument to encourage disproportionate recruitment in some areas beyond that which prevalence would suggest. It is important to note that there are other factors beyond prevalence to consider when judging the external validity of applied clinical health research, and recruiting in high prevalence areas may still be prone to selection bias.

Although there are significant potential benefits to ensuring that research populations represent the underlying population of need, recruitment in areas which have not traditionally been active in recruitment may be more costly (for example, if there is less infrastructure). This may have consequences for the volume, quality and rapidity of research delivery. Although high internal and external validity remains the goal of applied clinical research, these requirements can be in competition (especially in the context of limited resources) and it is difficult to judge the relative impact of reductions in internal and external validity.

## Conclusions

These findings could act as a stimulus to developing a broader understanding of what it means for research ‘to be conducted with and in the populations most affected’ [14] and the development of indicators to assess the fit.

Perhaps more importantly, these data could support ongoing assessment of policies which aim to encourage better fit between recruitment and disease burden. Analyses of redistribution using all data at the level of the LCRNs may provide the most reliable estimate of the amount of redistribution required. This would suggest that around 12% of the current recruitment activity could be modified to align better with disease prevalence, although we expect that this would be a lower bound, reflecting the limited granularity of data in the analyses at the LCRN level. Although further modelling is required to understand these relationships, there is also a need to understand *how* better alignment might be achieved. Recruitment remains an ongoing problem [4, 38], and the evidence about how best to improve recruitment is limited [39, 40]. There is a need to explore what sorts of interventions can enable better realignment (such as changes in how studies are funded, changes to network organisation, or by modifying incentives for research and clinical teams). The NIHR Clinical Research Network in England is in a unique position to be able to assess the relationship between recruitment and prevalence of disease. The experience in England may have implications for health care and research funding organisations worldwide.

## Supplementary information

**Additional file 1: Figure S1a-S1f.** Geographical distribution of recruitment. **Figures S2a-S2i.** Bar chart of rate of recruitment at different levels of disease prevalence. **Figures S3a-S3i.** Concentration curves. **Table S4.** Univariate regression analyses – effects of time on redistribution index.

## Data Availability

Data from the QOF are publically available from NHS Digital. Data on recruitment activity are property of the NIHR CRN.

## Abbreviations

NIHR: National Institute of Health Research
CRN: Clinical Research Network
LCRN: Local Clinical Research Networks
CRNCC: Clinical Research Network Coordinating Centre
CCG: Clinical commissioning group
QOF: Quality and Outcomes Framework

## References

[1] Downing A, Morris EJA, Corrigan N, Sebag-Montefiore D, Finan PJ, Thomas JD, Chapman M, Hamilton R, Campbell H, Cameron D (2017). High hospital research participation and improved colorectal cancer survival outcomes: a population-based study. Gut.

[2] Boaz A, Hanney S, Jones T, Soper B. Does the engagement of clinicians and organisations in research improve healthcare performance: a three stage review. BMJ open. 2015;5(12):e009415. 10.1136/bmjopen-2015-009415 PMC4680006 26656023

[3] Golsorkhi M, Steel R. Report of the patient research experience survey 2017/18. In: Clinical Research Network Coordinating Centre: NIHR; London 2018.

[4] McDonald A, Knight R, Campbell M, Entwistle V, Grant A, Cook J, Elbourne D, Francis D, Garcia J, Roberts I, et al. What influences recruitment to randomised controlled trials? A review of trials funded by two UK funding agencies. Trials. 2006;7:9. 10.1186/1745-6215-7-9 PMC1475627 16603070

[5] Fry A, Sudlow C, Adamska L, Allen NE, Doherty N, Collins R, Sprosen T, Littlejohns TJ (2017). Comparison of sociodemographic and health-related characteristics of UK biobank participants with those of the general population. Am J Epidemiol.

[6] Brown G, Marshall M, Bower P, Woodham A, Waheed W (2014). Barriers to recruiting ethnic minorities to mental health research: a systematic review. Int J Methods Psychiatr Res.

[7] Matthews P, Netto G, Besemer K (2012). 'Hard-to-Reach'or ‘easy-to-ignore’? A rapid review of place-based policies and equality.

[8] Fortin M, Smith S (2013). Improving the external validity of clinical trials: the case of multiple chronic conditions. J Comorb.

[9] Treweek S, Dryden R, McCowan C, Harrow A, Thompson AM (2015). Do participants in adjuvant breast cancer trials reflect the breast cancer patient population?. Eur J Cancer.

[10] Macleod M, Michie S, Roberts I, Dirnagl U, Chalmers I, Ioannidis J, Al Shahi Salman R, Chan A, Glasziou P (2014). Biomedical research: increasing value, reducing waste. Lancet.

[11] Darbyshire J (2008). The UK clinical research network-building a world-class infrastructure for clinical research. Rheumatology.

[12] Davies SC, Walley T, Smye S, Cotterill L, Whitty CJM (2016). The NIHR at 10: transforming clinical research. Clin Med.

[13] Rothwell P (2005). External validity of randomised controlled trials: to whom do the results of this trial apply?. Lancet.

[14] Whitty C, Wood L (2017). Shaping the future of NIHR. Department of Health and NIHR.

[15] Barnett B, Mercer S, Norbury M, Watt G, Wyke S, Guthrie B (2012). The epidemiology of multimorbidity in a large cross-sectional dataset: implications for health care, research and medical education. Lancet.

[16] Office for National Statistics. Disability in England and Wales: 2011 and comparison with 2001: Office for National Statistics; 2013. https://www.ons.gov.uk/census/2011census/censusanalysisindex. Accessed 28 Mar 2019.

[17] Travers J, Marsh S, Caldwell B, Williams M, Aldington S, Weatherall M, Shirtcliffe P, Beasley R (2007). External validity of randomized controlled trials in COPD. Respir Med.

[18] Saunders C, Byrne CD, Guthrie B, Lindsay RS, McKnight JA, Philip S, Sattar N, Walker JJ, Wild SH, on behalf of the Scottish Diabetes Research Network Epidemiology G (2013). External validity of randomized controlled trials of glycaemic control and vascular disease: how representative are participants?. Diabet Med.

[19] Walters SJ, Bonacho dos Anjos Henriques-Cadby I, Bortolami O, Flight L, Hind D, Jacques RM, Knox C, Nadin B, Rothwell J, Surtees M, et al. Recruitment and retention of participants in randomised controlled trials: a review of trials funded and published by the United Kingdom Health Technology Assessment Programme. BMJ Open. 2017;7(3). 10.1136/bmjopen-2016-015276 PMC5372123 28320800

[20] Buchan IE, Kontopantelis E, Sperrin M, Chandola T, Doran T (2017). North-south disparities in English mortality1965–2015: longitudinal population study. J Epidemiol Community Health.

[21] Kontopantelis E, Buchan I, Webb RT, Ashcroft DM, Mamas MA, Doran T (2018). Disparities in mortality among 25-44-year-olds in England: a longitudinal, population-based study. Lancet Public Health.

[22] Shiell A, Hawe P, Gold L (2008). Complex interventions or complex systems? Implications for health economic evaluation. BMJ.

[23] Pawson R, Greenhalgh T, Harvey G, Walshe K (2005). Realist review - a new method of systematic review designed for complex policy interventions. J Health Serv Res Policy.

[24] Rothwell PM (2006). Factors that can affect the external validity of randomised controlled trials. PLoS Clin Trials.

[25] NHS Digital. Quality and Outcomes Framework, achievement, prevalence and exceptions data, 2017–18: NHS Digital; 2018. Available at: https://digital.nhs.uk/data-and-information/publications/statistical/quality-and-outcomes-framework-achievement-prevalence-and-exceptions-data/2017-18.

[26] Olier I, Springate DA, Ashcroft DM, Doran T, Reeves D, Planner C, Reilly S, Kontopantelis E. Modelling conditions and health care processes in electronic health records: an application to severe mental illness with the Clinical Practice Research Datalink. PLoS One. 2016;11(2). 10.1371/journal.pone.0146715 PMC4769302 26918439

[27] MQ (2018). UK Mental Health Research Funding 2014–2017.

[28] Woelbert E, Kirtley A, Balmer N, Dix S. How much is spent on mental health research: developing a system for categorising grant funding in the UK. Lancet Psychiatry. 2019;6(5):445-52. 10.1016/S2215-0366(19)30033-1 30824371

[29] Koolman X, van Doorslaer E (2004). On the interpretation of a concentration index of inequality. Health Econ.

[30] O'donnell, Owen, Eddy Van Doorslaer, Adam Wagstaff, and Magnus Lindelow. Analyzing health equity using household survey data: a guide to techniques and their implementation. The World Bank, 2007.

[31] Ozdemir BA, Karthikesalingam A, Sinha S, Poloniecki JD, Hinchliffe RJ, Thompson MM, Gower JD, Boaz A, Holt PJE (2015). Research activity and the association with mortality. PLoS One.

[32] Parekh AK, Barton MB (2010). The challenge of multiple comorbidity for the US health care system. JAMA.

[33] Lehnert T, Heider D, Leicht H, Heinrich S, Corrieri S, Luppa M, Riedel-Heller S, König H-H (2011). Review: health care utilization and costs of elderly persons with multiple chronic conditions. Med Care Res Rev.

[34] James SL, Abate D, Abate KH, Abay SM, Abbafati C, Abbasi N, Abbastabar H, Abd-Allah F, Abdela J, Abdelalim A (2018). Global, regional, and national incidence, prevalence, and years lived with disability for 354 diseases and injuries for 195 countries and territories, 1990-2017: a systematic analysis for the Global Burden of Disease Study 2017. Lancet.

[35] Newton JN, Briggs ADM, Murray CJL, Dicker D, Foreman KJ, Wang H, Naghavi M, Forouzanfar MH, Ohno SL, Barber RM (2015). Changes in health in England, with analysis by English regions and areas of deprivation, 1990–2013: a systematic analysis for the Global Burden of Disease Study 2013. Lancet.

[36] Prados-Torres A, Calderón-Larrañaga A, Hancco-Saavedra J, Poblador-Plou B, van den Akker M (2014). Multimorbidity patterns: a systematic review. J Clin Epidemiol.

[37] Piette J, Kerr E (2006). The impact of comorbid chronic conditions on diabetes care. Diabetes Care.

[38] Sully B, Julious S, Nicholl J (2013). A reinvestigation of recruitment to randomised, controlled, multicenter trials: a review of trials funded by two UK funding agencies. Trials.

[39] Treweek S, Pitkethly M, Cook J, Fraser C, Mitchell E, Sullivan F, Jackson C, Taskila TK, Gardner H. Strategies to improve recruitment to randomised trials. Cochrane Database Syst Rev. 2018;2. 10.1002/14651858.MR000013.pub6 PMC7078793 29468635

[40] Elliott D, Husbands S, Hamdy FC, Holmberg L, Donovan JL (2017). Understanding and improving recruitment to randomised controlled trials: qualitative research approaches. Eur Urol.

