# Supplementary material for: Is health research undertaken where the burden of disease is greatest? Observational study of geographical inequalities in recruitment to research in England 2013–2018
Source: BMC Med. 2020 May 18;18:133. doi: 10.1186/s12916-020-01555-4 (PMC7232839; doi:10.1186/s12916-020-01555-4)
Supplement: Supplementary file 1 — Additional file 1: Figure S1a-S1f. Geographical distribution of recruitment. Figures S2a-S2i. Bar chart of rate of recruitment at different levels of disease prevalence. Figures S3a-S3i. Concentration curves. Table S4. Univariate regression analyses – effects of time on redistribution index. [file 12916_2020_1555_MOESM1_ESM.docx]

**Appendix Figure 1a Geographical distribution of recruitment per 1000 people across the 195 CCGs in England (all conditions, primary care data, CCG level)**

Darker areas have higher levels of recruitment per 1000 people (attribute classification method in 4 quantiles)


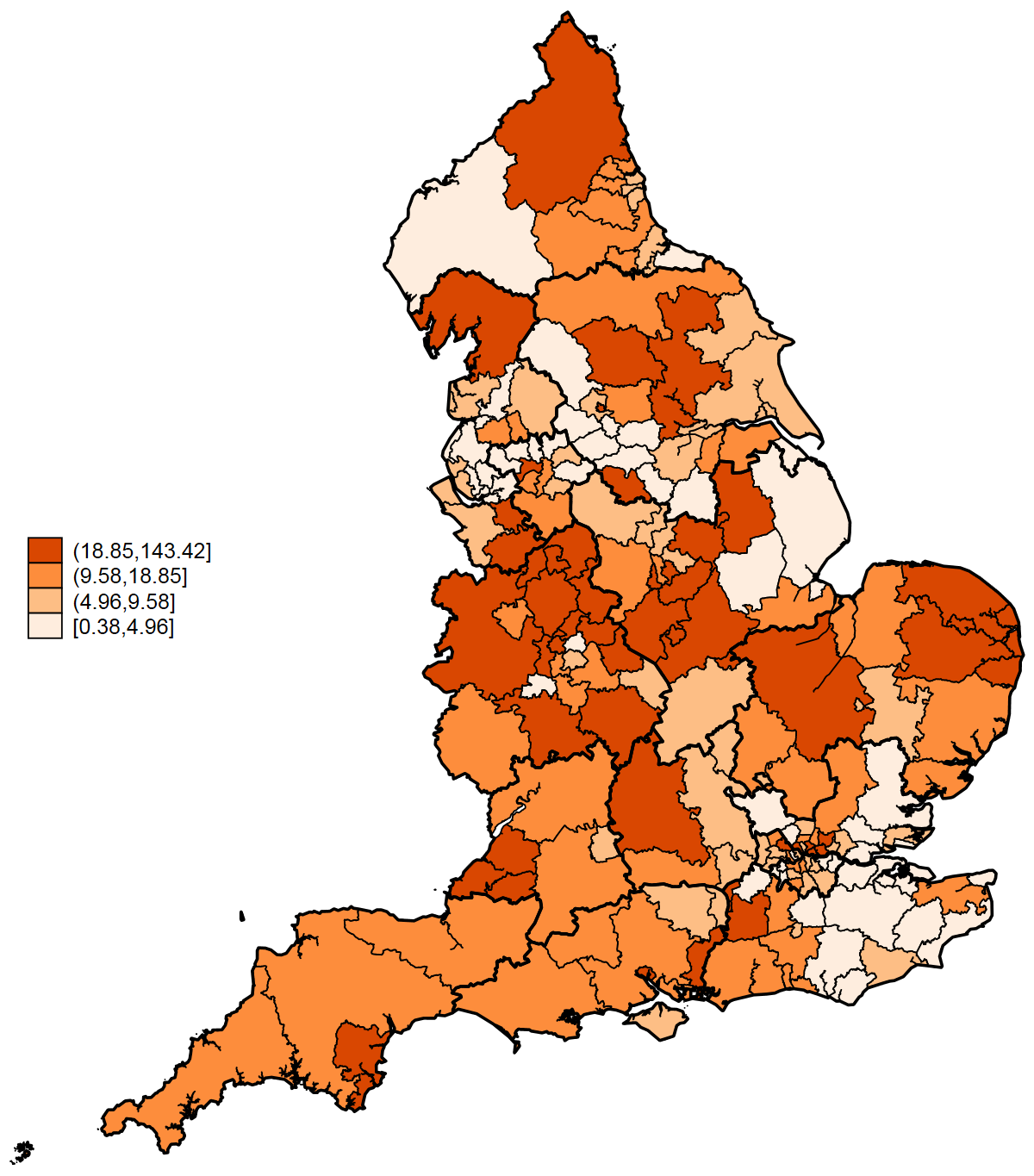


**Appendix Figure 1b Geographical distribution of recruitment per 1000 people across the 195 CCGs in England (all conditions, all data, CCG level)**

Darker areas have higher levels of recruitment per 1000 people (attribute classification method in 4 quantiles)


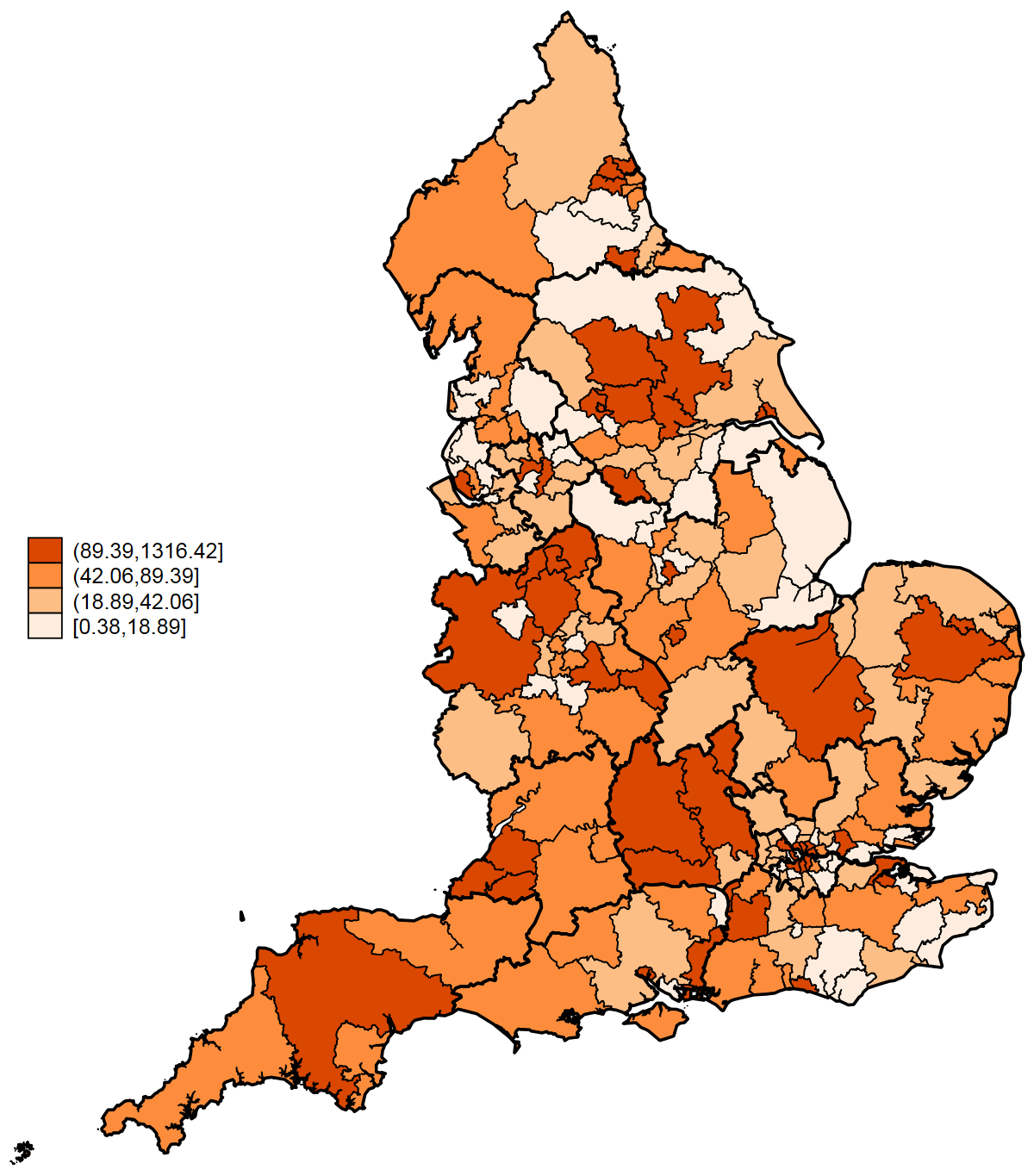


**Appendix Figure 1c Geographical distribution of recruitment per 1000 people across the 195 CCGs in England (mental health, primary care data, CCG level)**

Darker areas have higher levels of recruitment per 1000 people (attribute classification method in 4 quantiles)


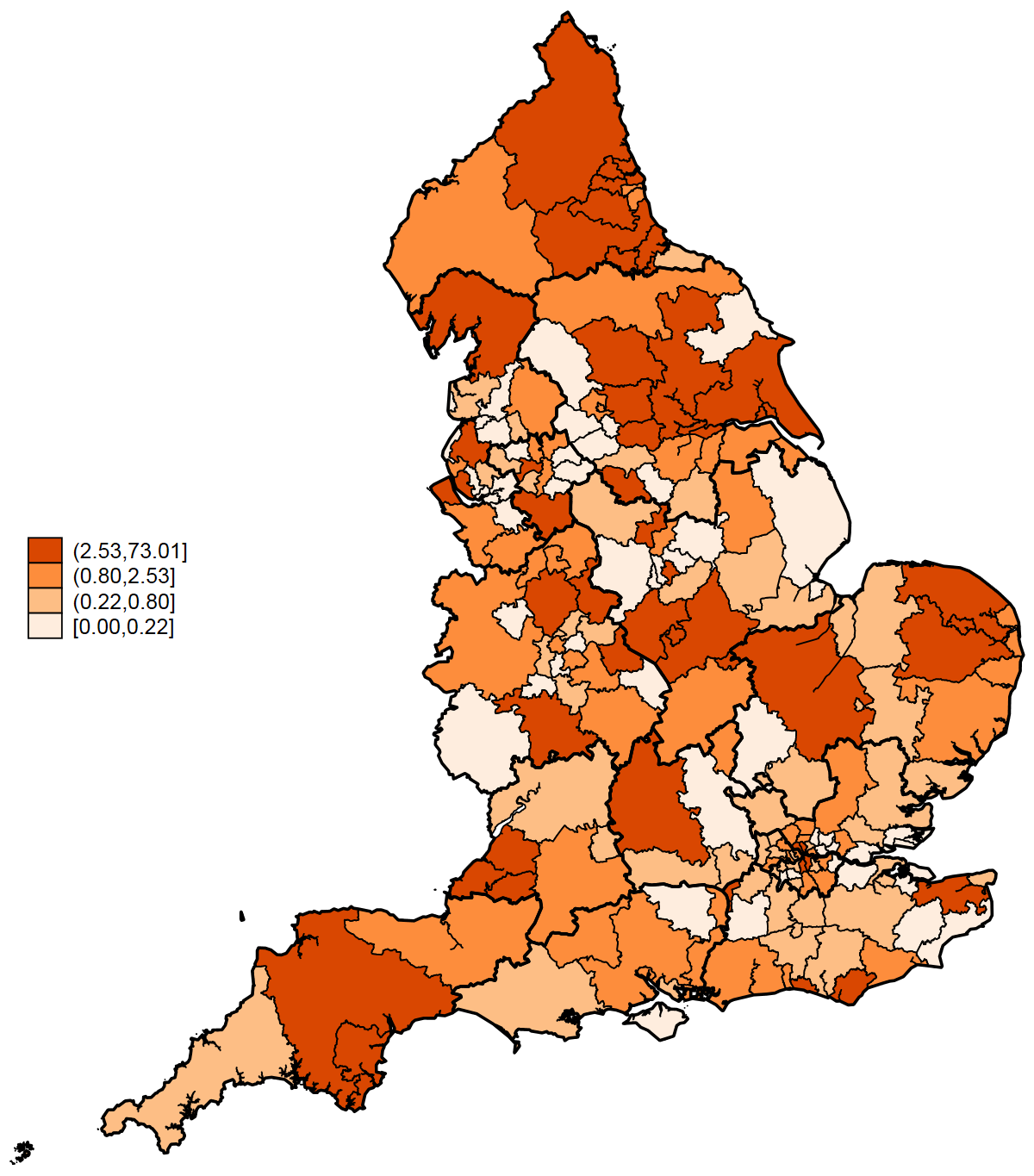


**Appendix Figure 1d Geographical distribution of recruitment per 1000 people across the 195 CCGs in England (mental health, all data, CCG level)**

Darker areas have higher levels of recruitment per 1000 people (attribute classification method in 4 quantiles)


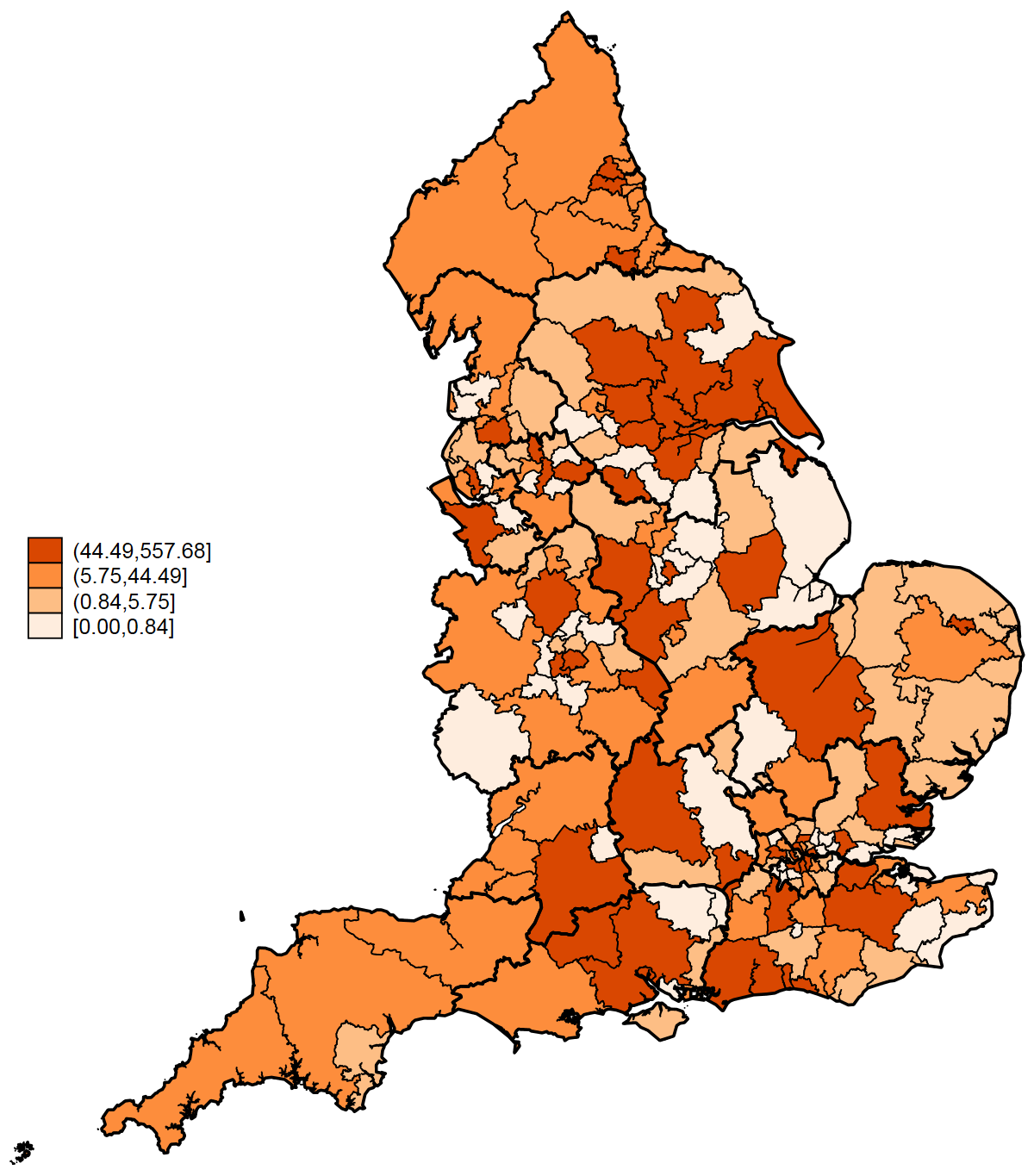


**Appendix Figure 1e Geographical distribution of recruitment per 1000 people across the 195 CCGs in England (diabetes, primary care data, CCG level)**

Darker areas have higher levels of recruitment per 1000 people (attribute classification method in 4 quantiles)


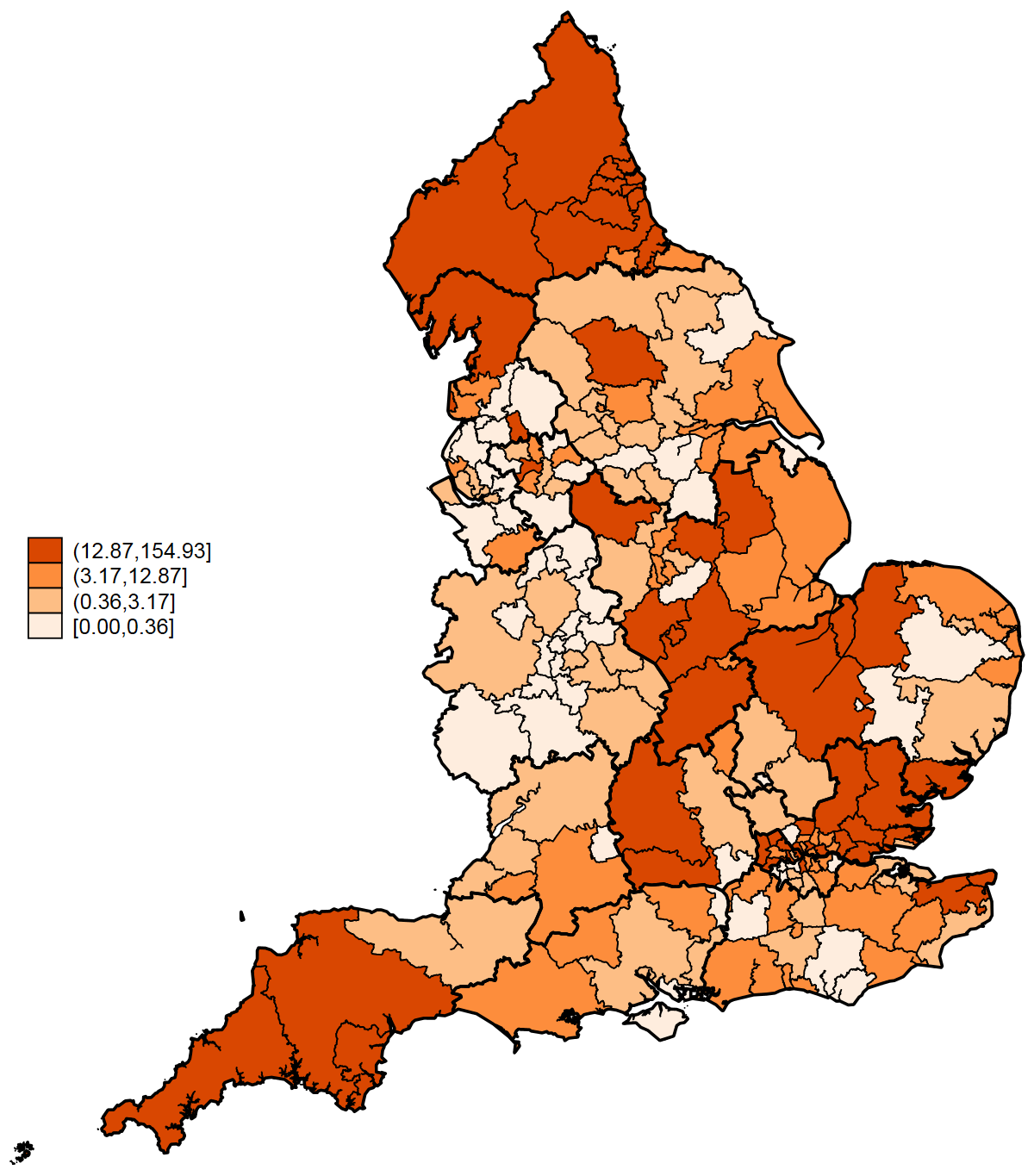


**Appendix Figure 1f Geographical distribution of recruitment per 1000 people across the 195 CCGs in England (diabetes, all data, CCG level)**

Darker areas have higher levels of recruitment per 1000 people (attribute classification method in 4 quantiles)
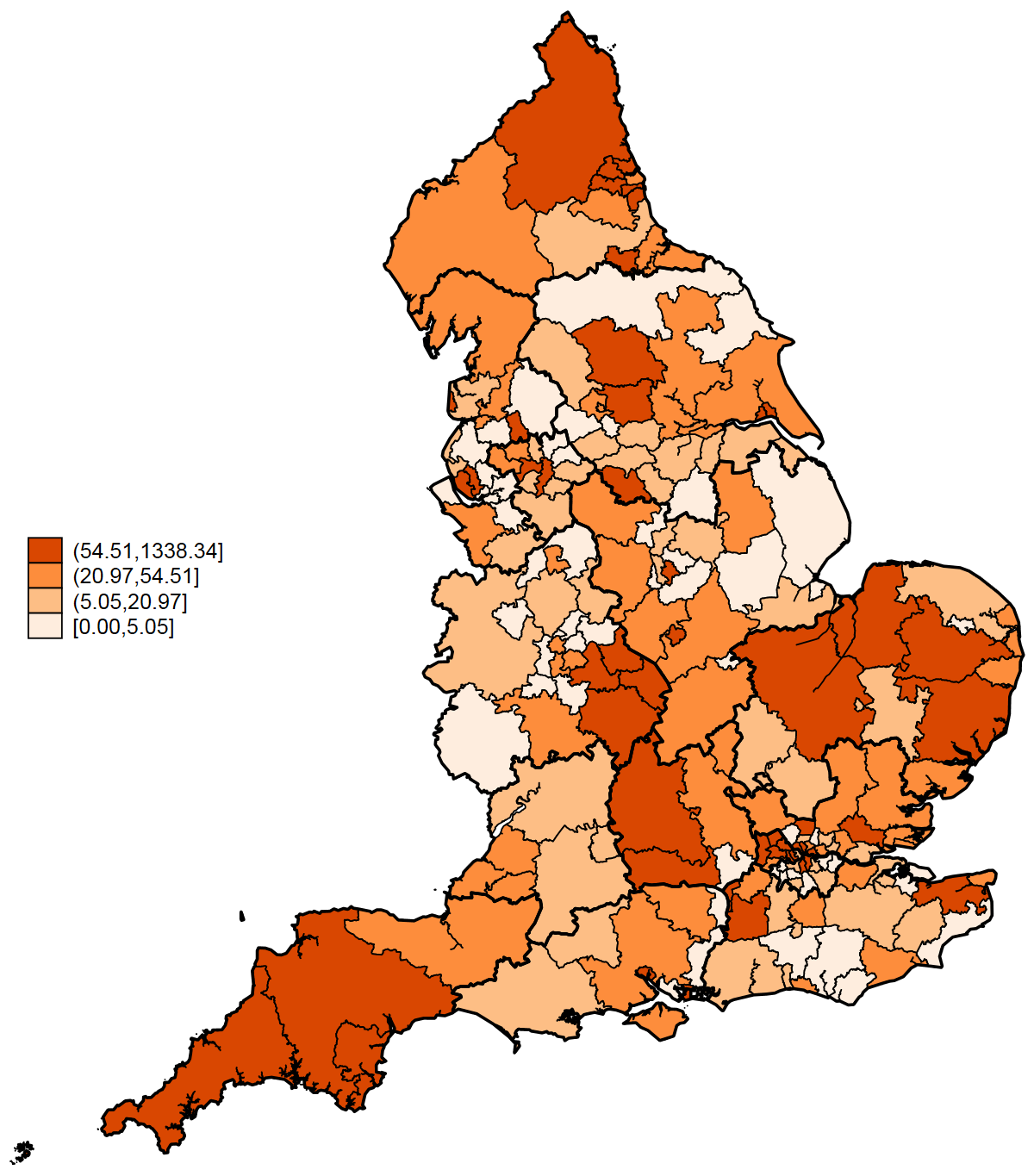


**Appendix Figure 2a Bar chart of rate of recruitment at different levels of disease prevalence (all conditions, all data, CCG level, CCG quintiles ranked by prevalence low-> high)**

**
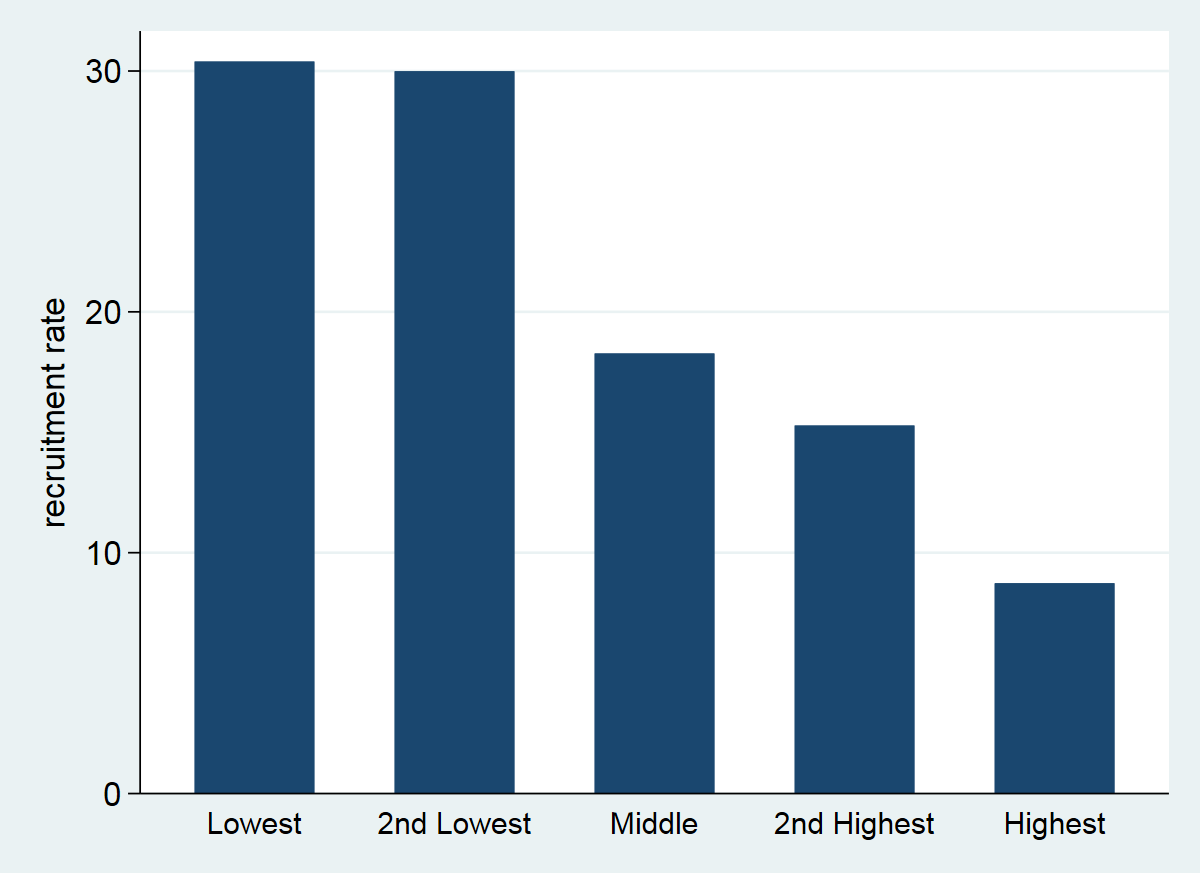
**

**Appendix Figure 2b Bar chart of rate of recruitment at different levels of disease prevalence (all conditions, all data, LCRN level, LCRNs ranked by prevalence low-> high)**

**
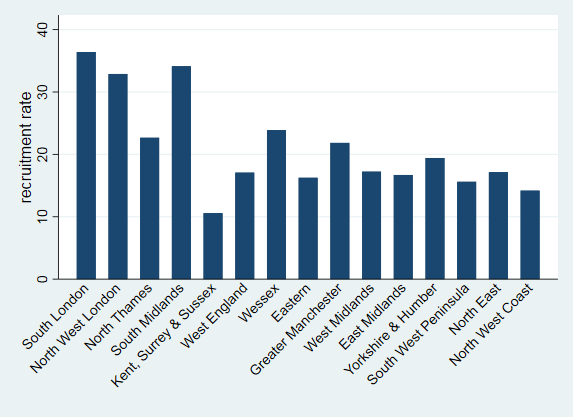
**

**Appendix Figure 2c Bar chart of rate of recruitment at different levels of disease prevalence (all conditions, primary care data, CCG level, CCG quintiles ranked by prevalence low-> high)**


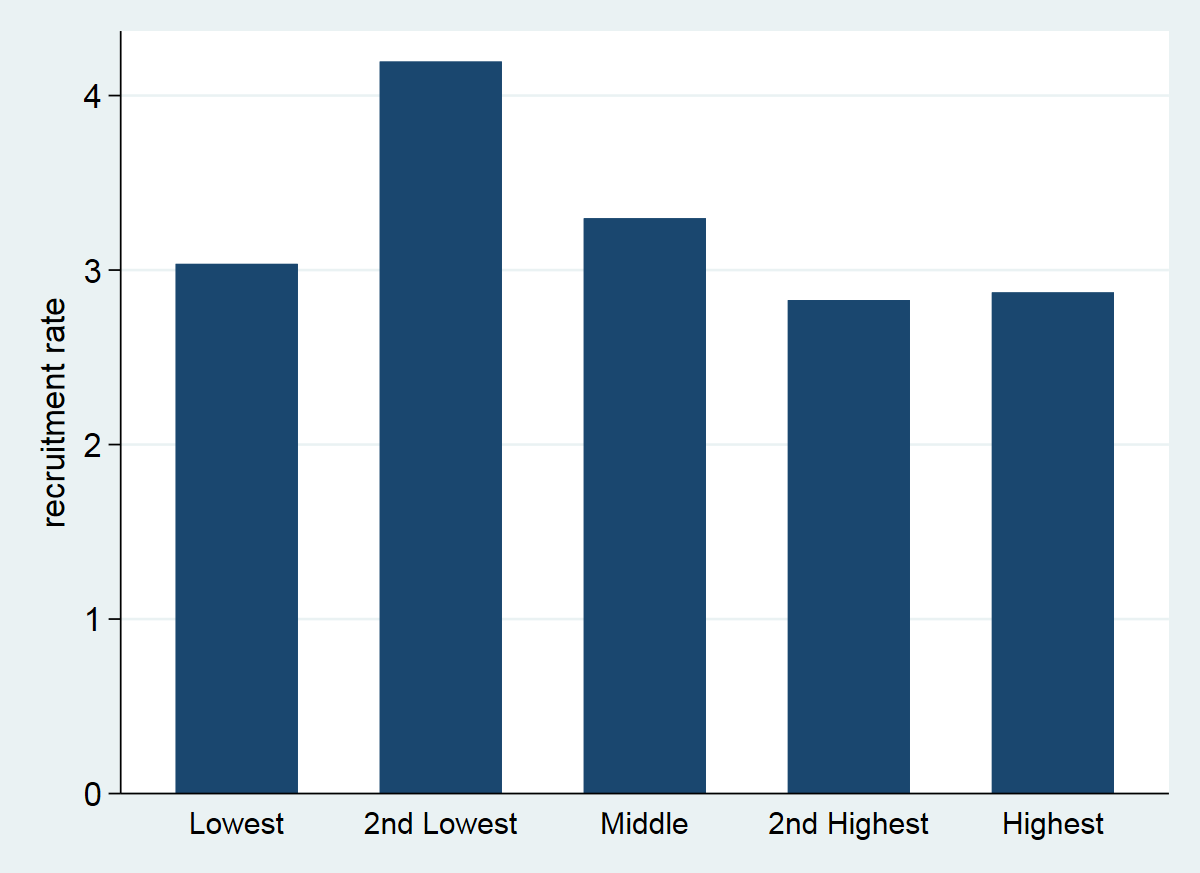


**Appendix Figure 2a Bar chart of rate of recruitment at different levels of disease prevalence (mental health, all data, CCG level, CCG quintiles ranked by prevalence low-> high)**

**
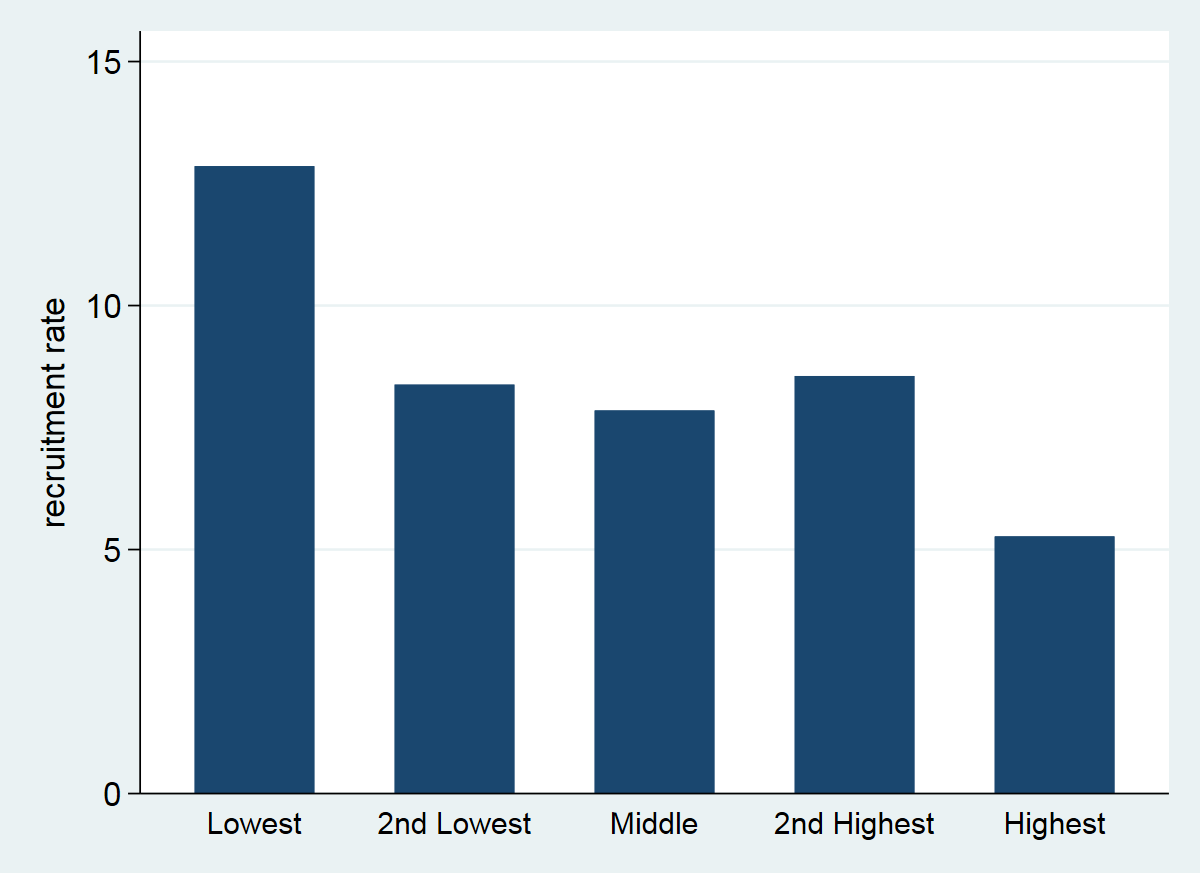
**

**Appendix Figure 2b Bar chart of rate of recruitment at different levels of disease prevalence (mental health, all data, LCRN level, LCRNs ranked by prevalence low-> high)**


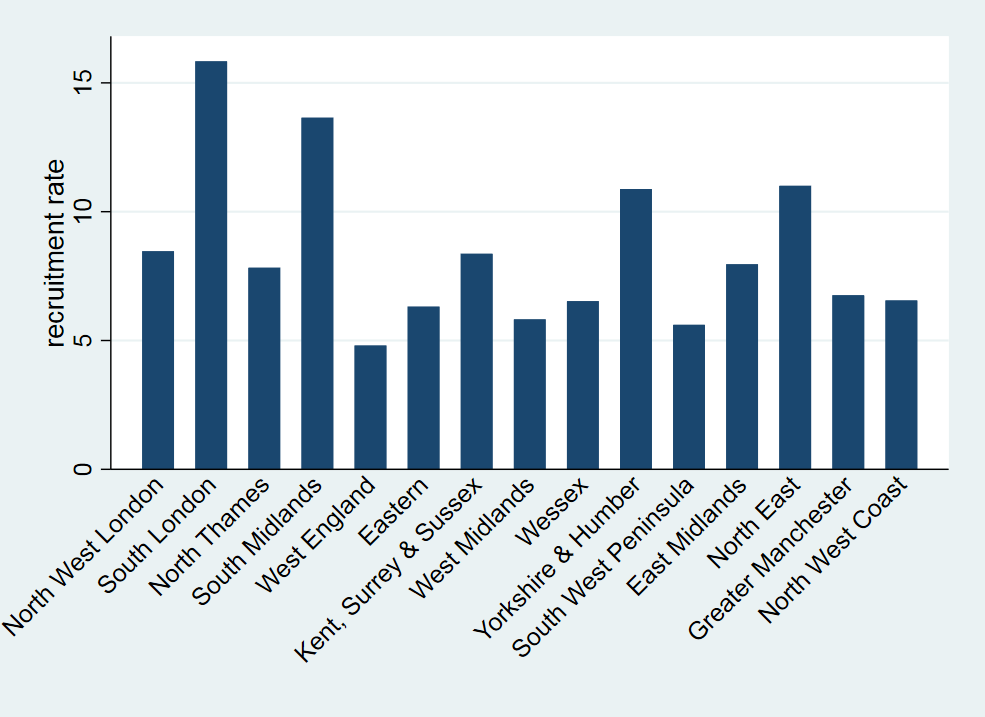


**Appendix Figure 2c Bar chart of rate of recruitment at different levels of disease prevalence (mental health, primary care data, CCG level, CCG quintiles ranked by prevalence low-> high)**


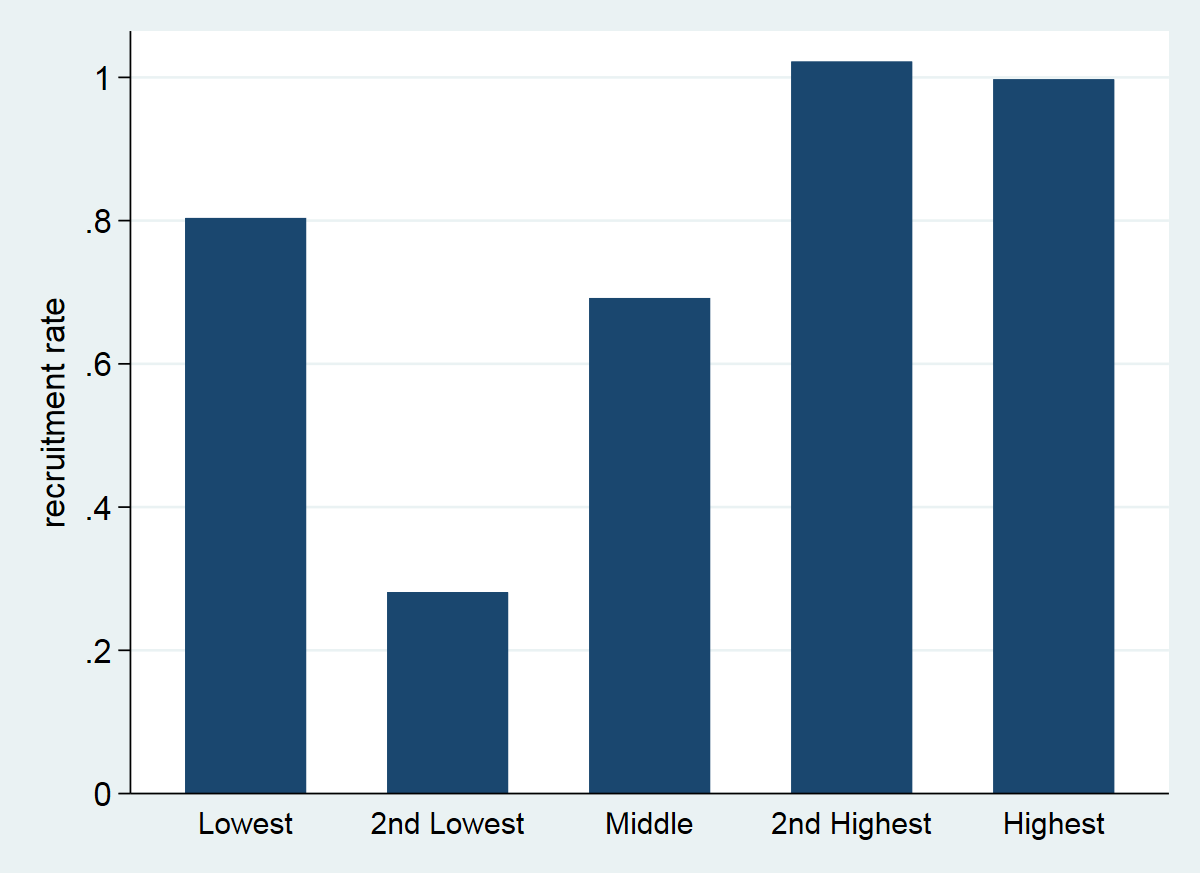


**Appendix Figure 2g Bar chart of rate of recruitment at different levels of disease prevalence (diabetes, all data, CCG level, CCG quintiles ranked by prevalence low-> high)**

**
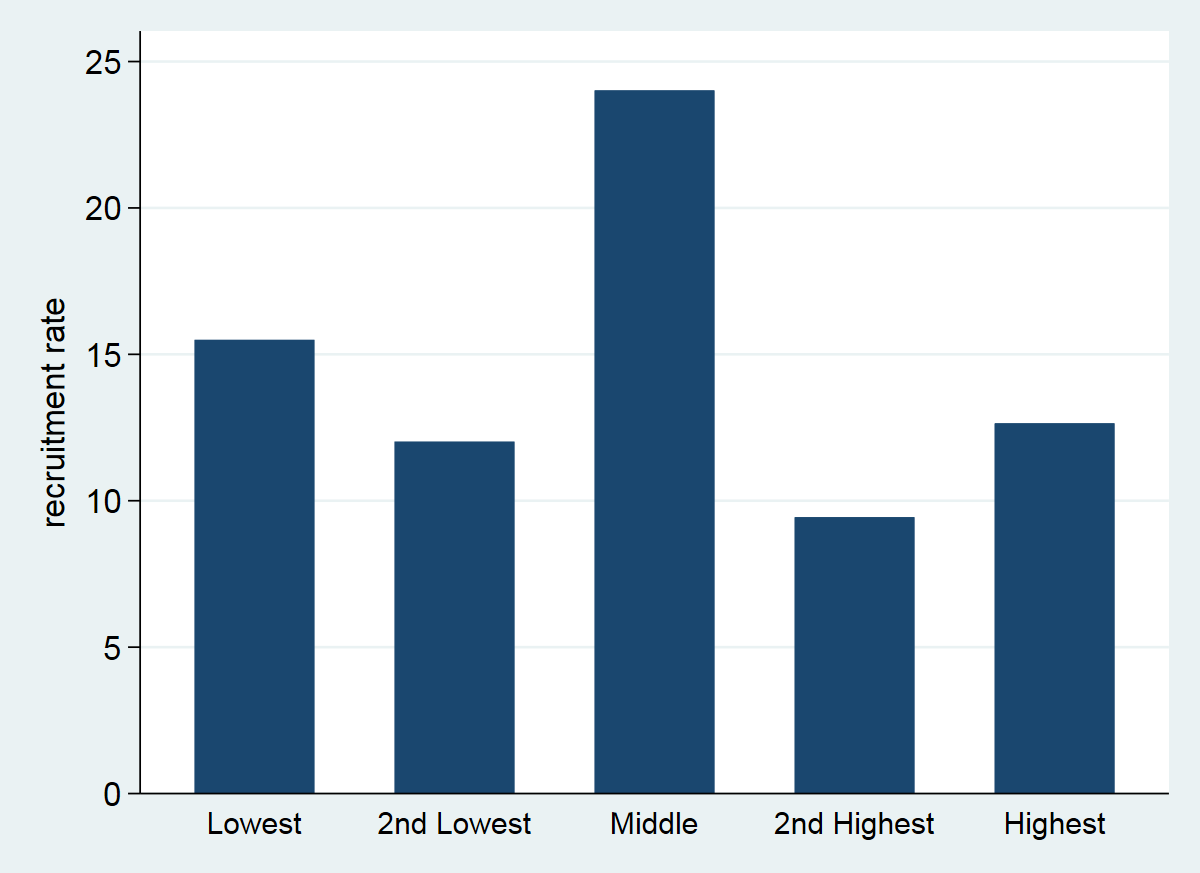
**

**Appendix Figure 2h Bar chart of rate of recruitment at different levels of disease prevalence (diabetes, all data, LCRN level, LCRNs ranked by prevalence low-> high)**

**
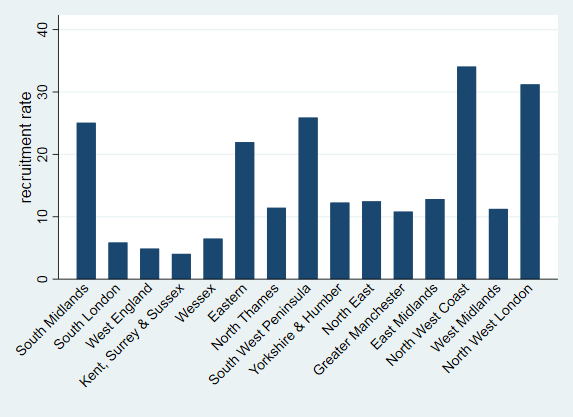
**

**Appendix Figure 2i Bar chart of rate of recruitment at different levels of disease prevalence (diabetes, primary care data, CCG level, CCG quintiles ranked by prevalence low-> high)**

**
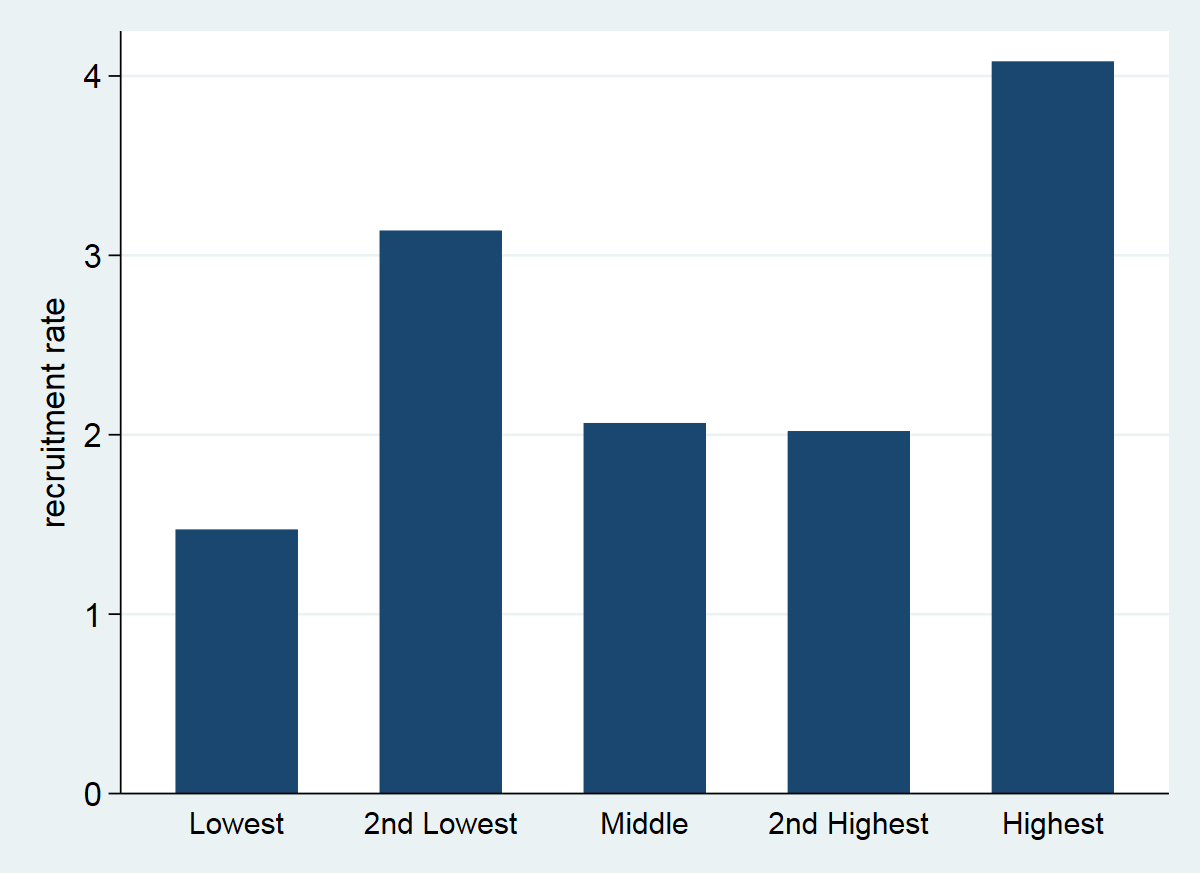
**

**Appendix Figure 3a Concentration curve (all conditions, all data, CCG level)**

**
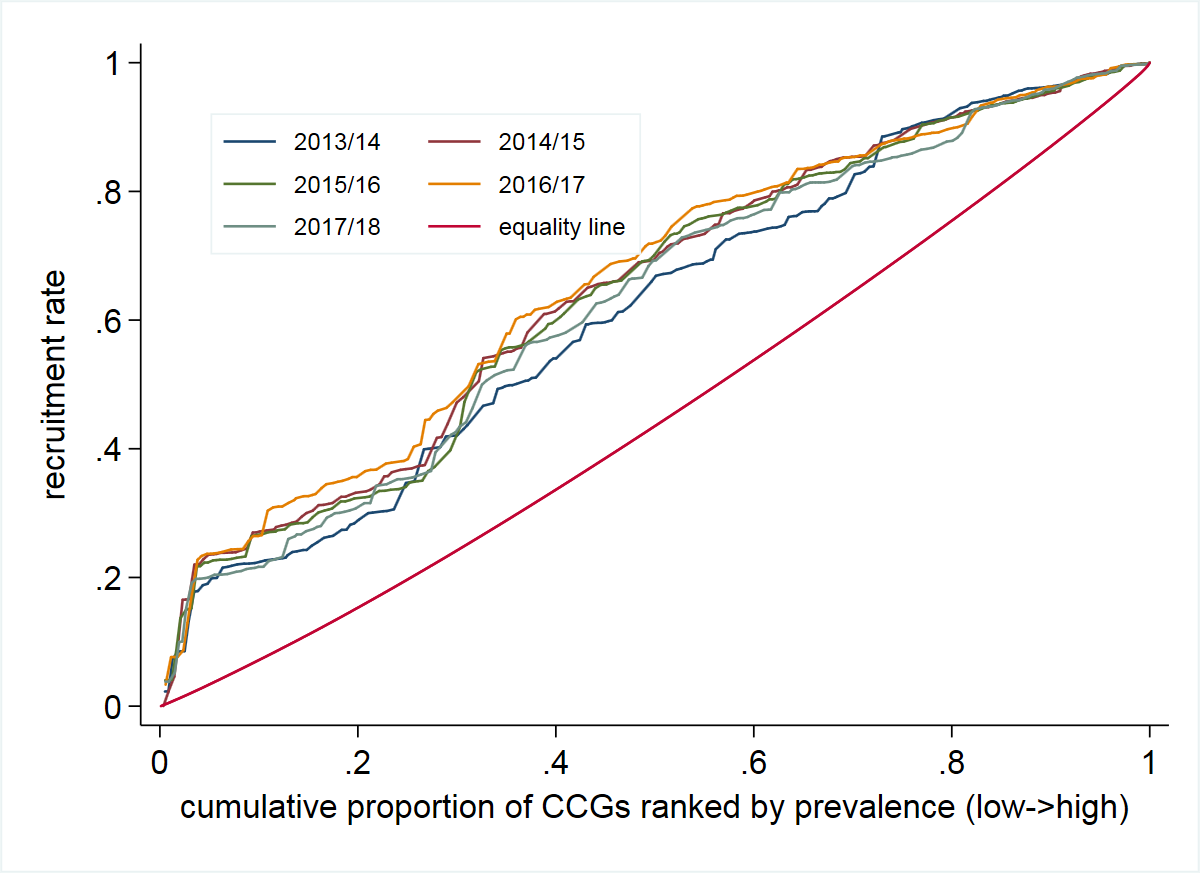
**

**Appendix Figure 3b Concentration curve (all conditions, all data, LCRN level)**

**
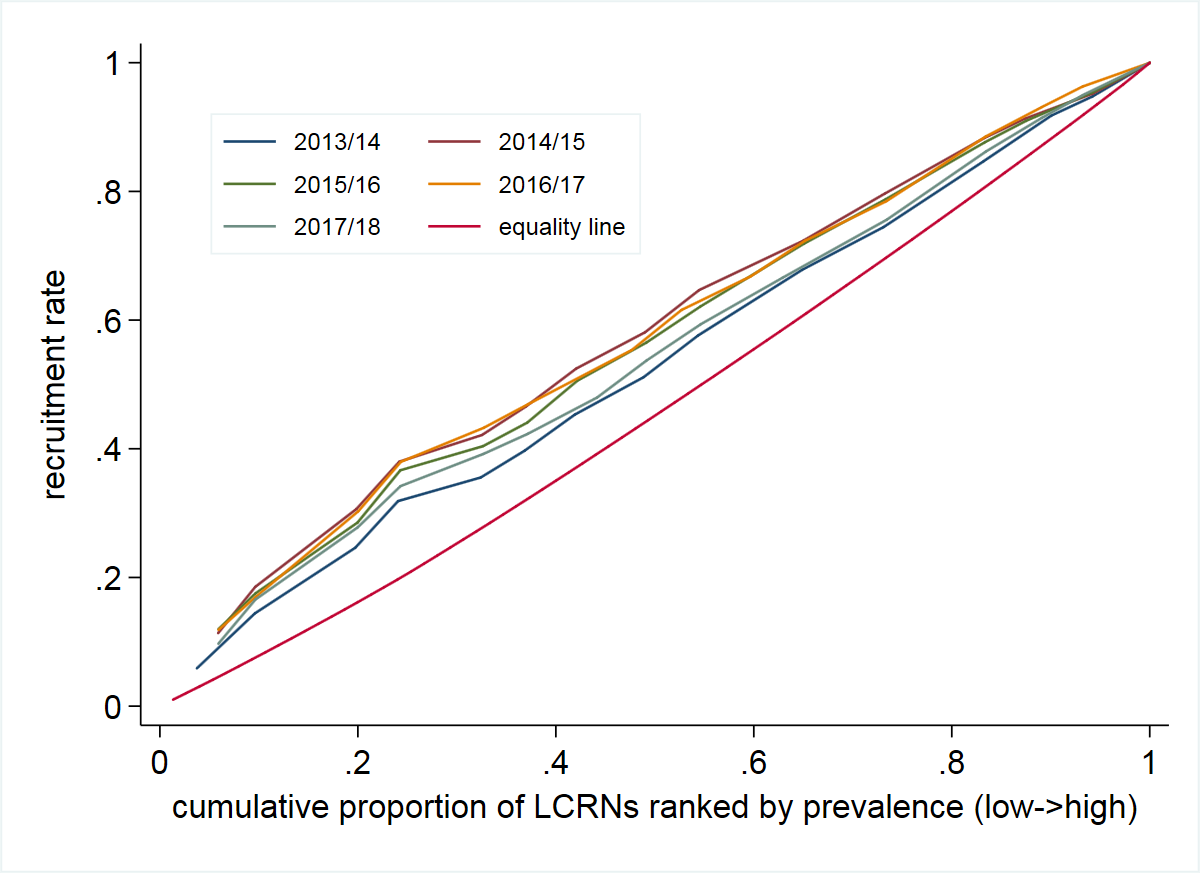
**

**Appendix Figure 3c Concentration curve (all conditions, primary care data, CCG level)**

**
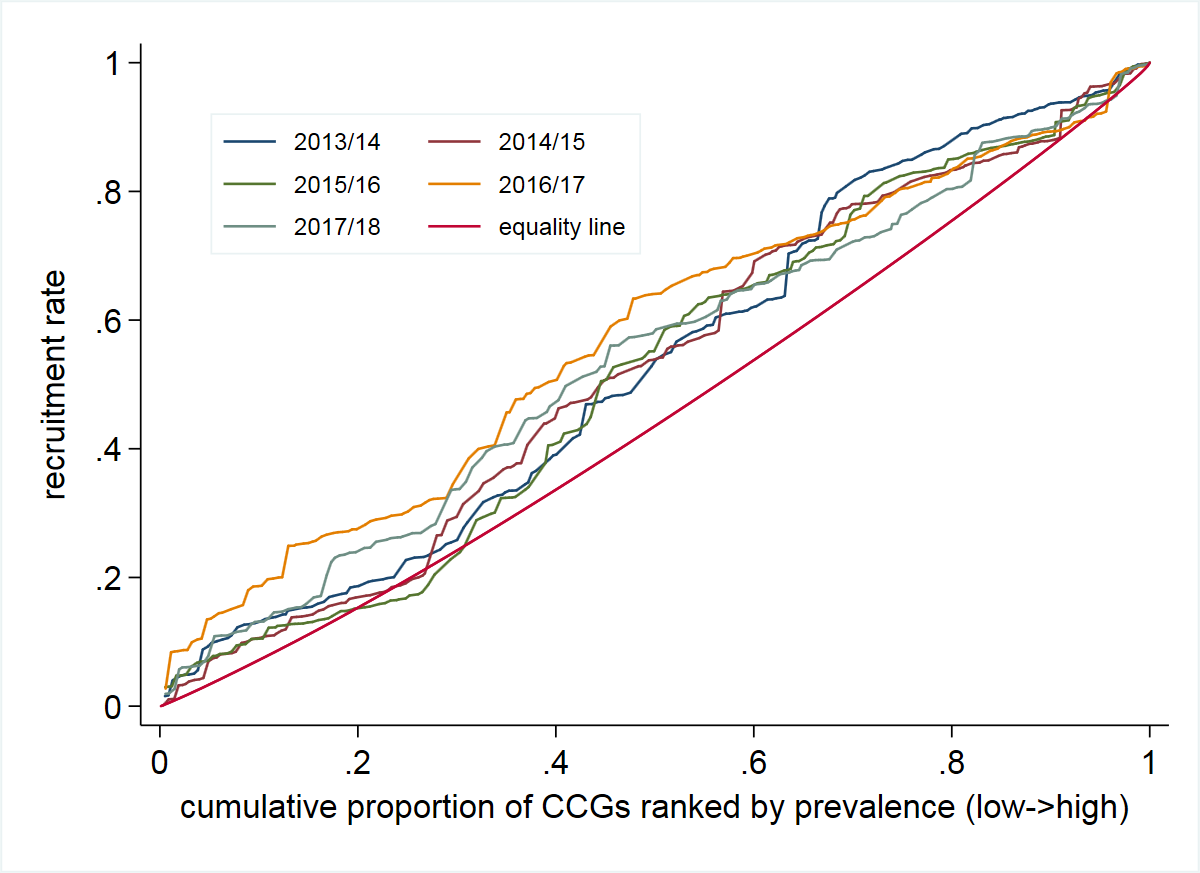
**

**Appendix Figure 3d Concentration curve (mental health, all data, CCG level)**

**
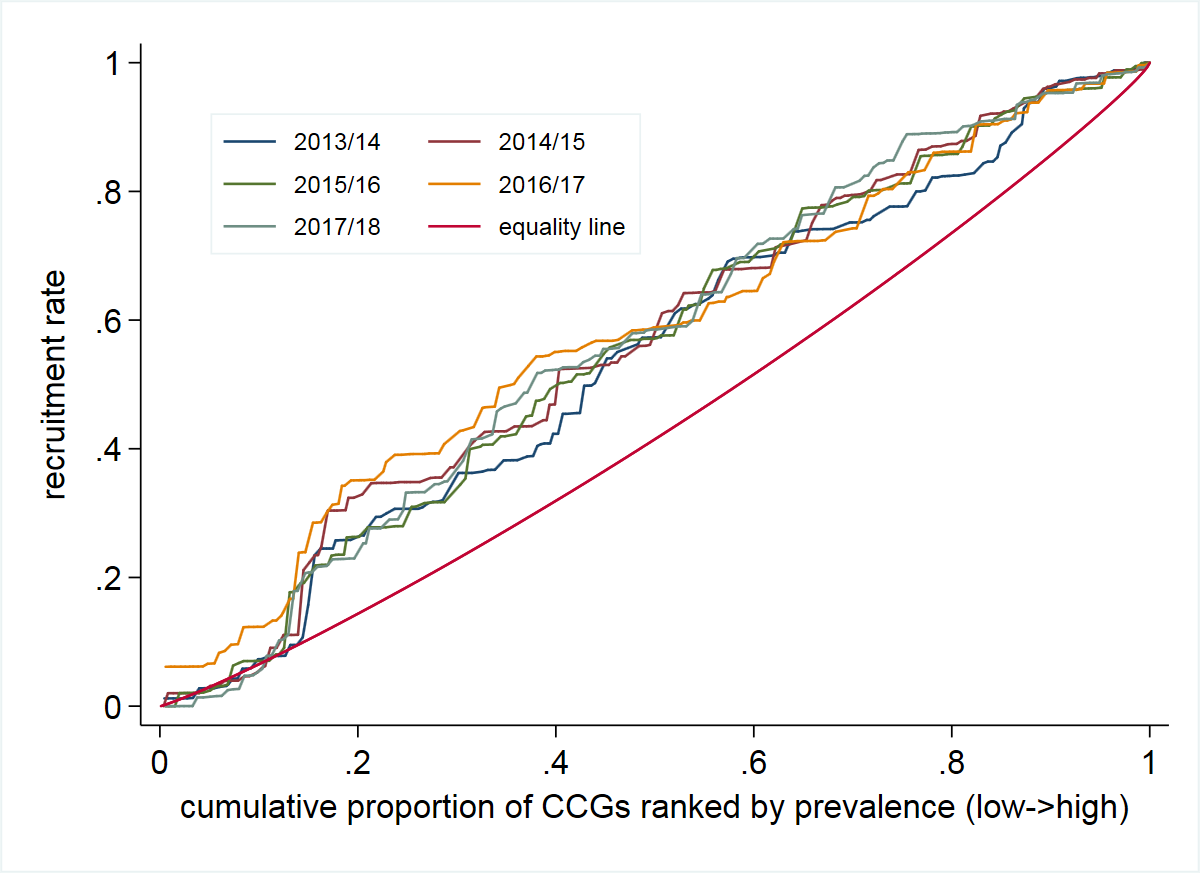
**

**Appendix Figure 3e Concentration curve (mental health, all data, LCRN level)**

**
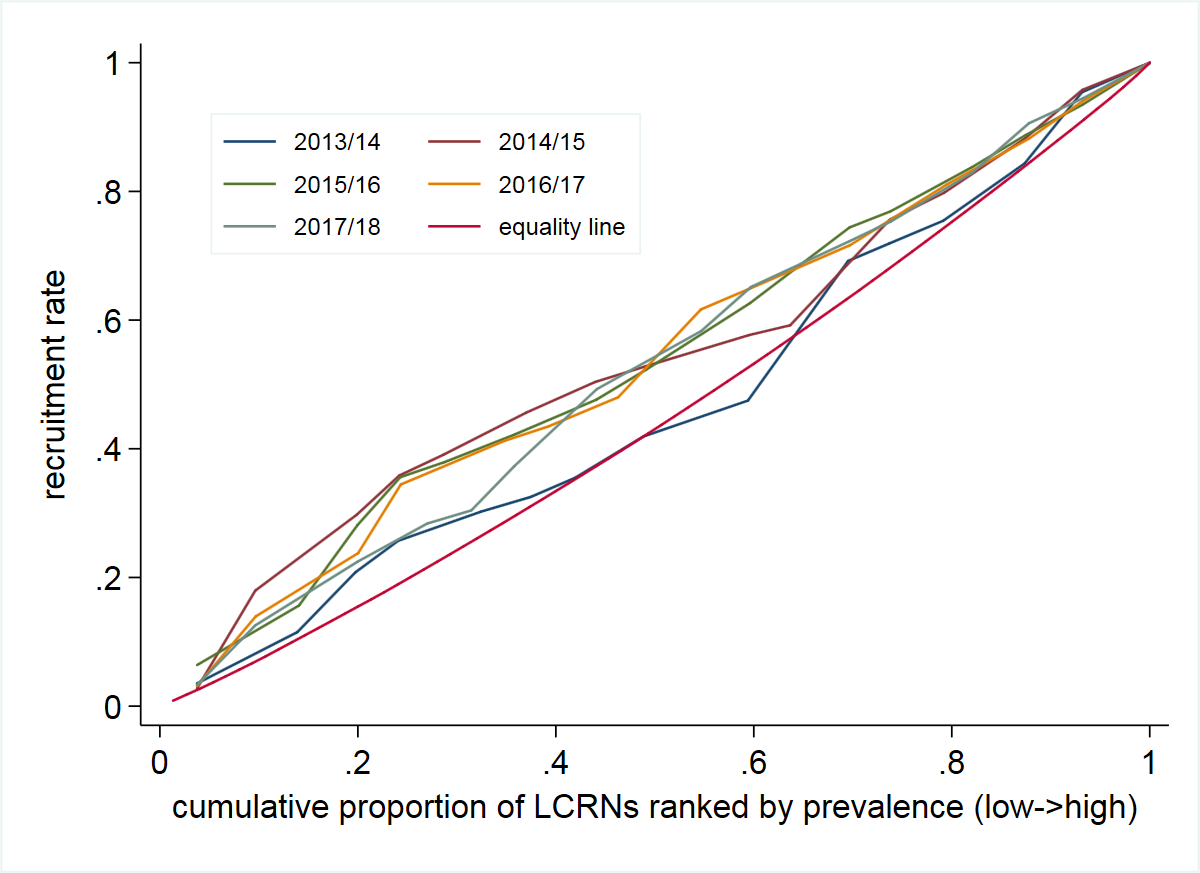
**

**Appendix Figure 3f Concentration curve (mental health, primary care data, CCG level)**

**
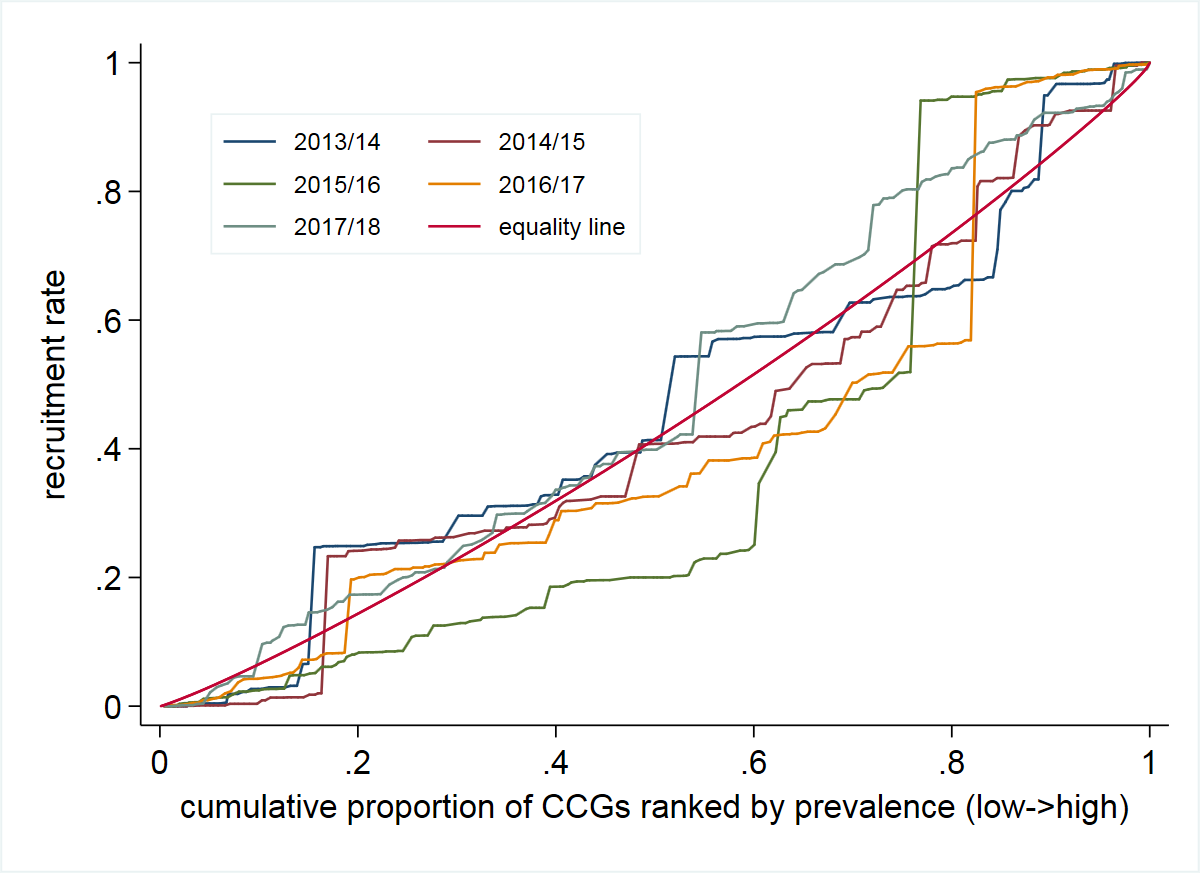
**

**Appendix Figure 3g Concentration curve (diabetes, all data, CCG level)**

**
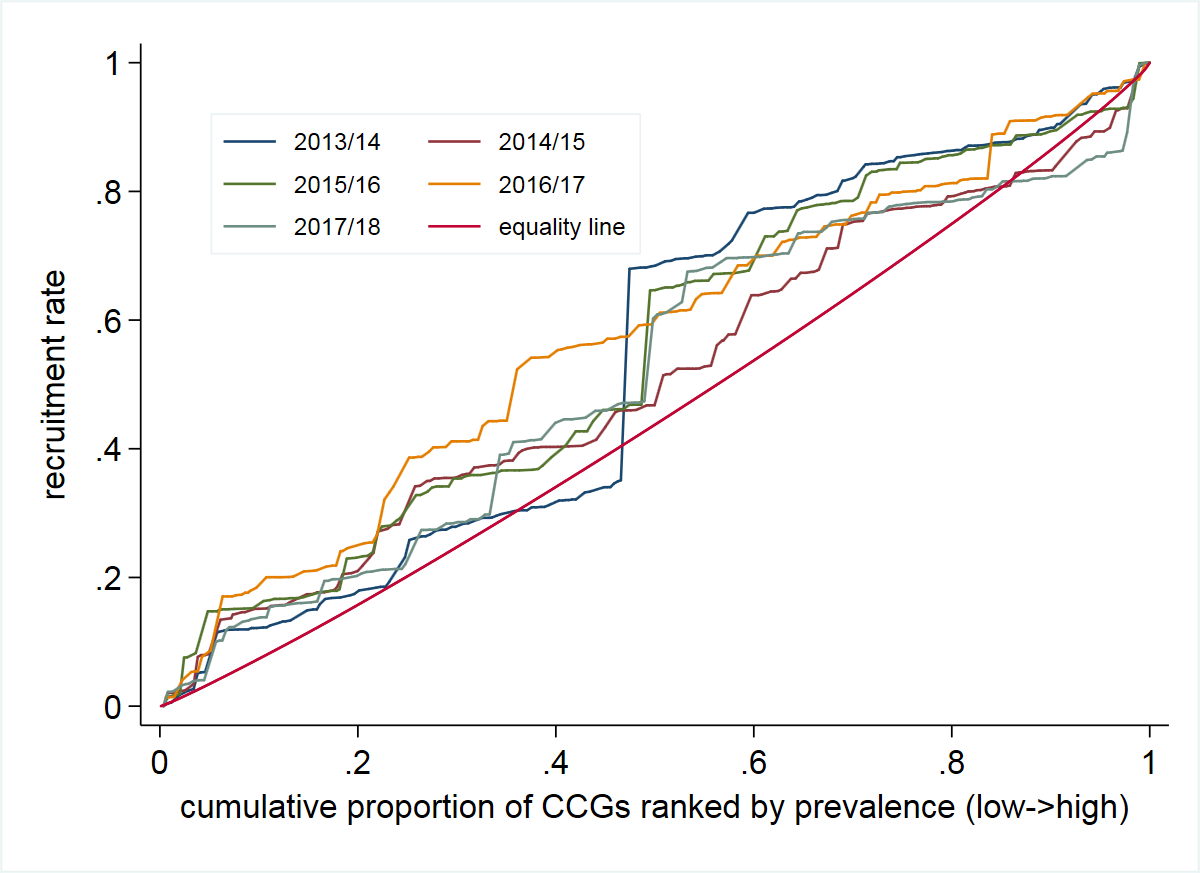
**

**Appendix Figure 3h Concentration curve (diabetes, all data, LCRN level)**

**
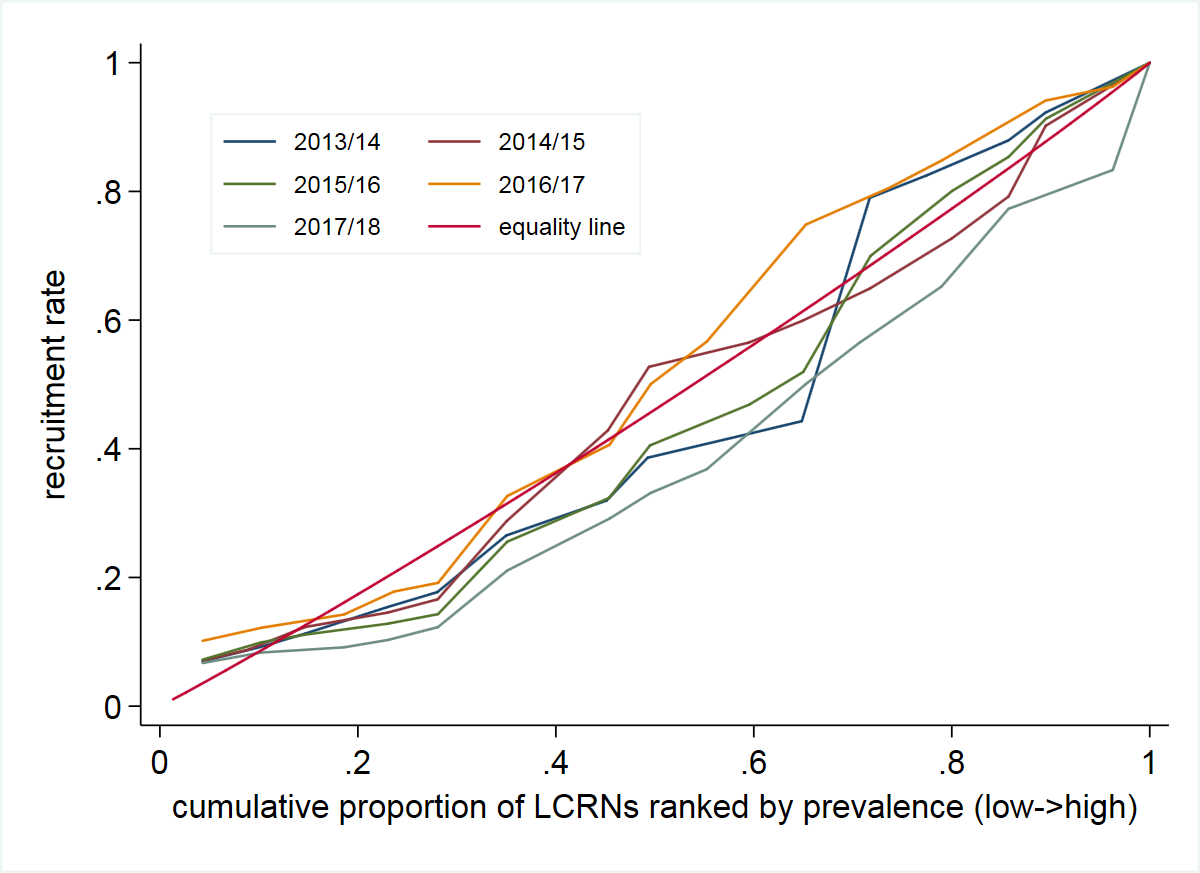
**

**Appendix Figure 3i Concentration curve (diabetes, primary care data, CCG level)**

**
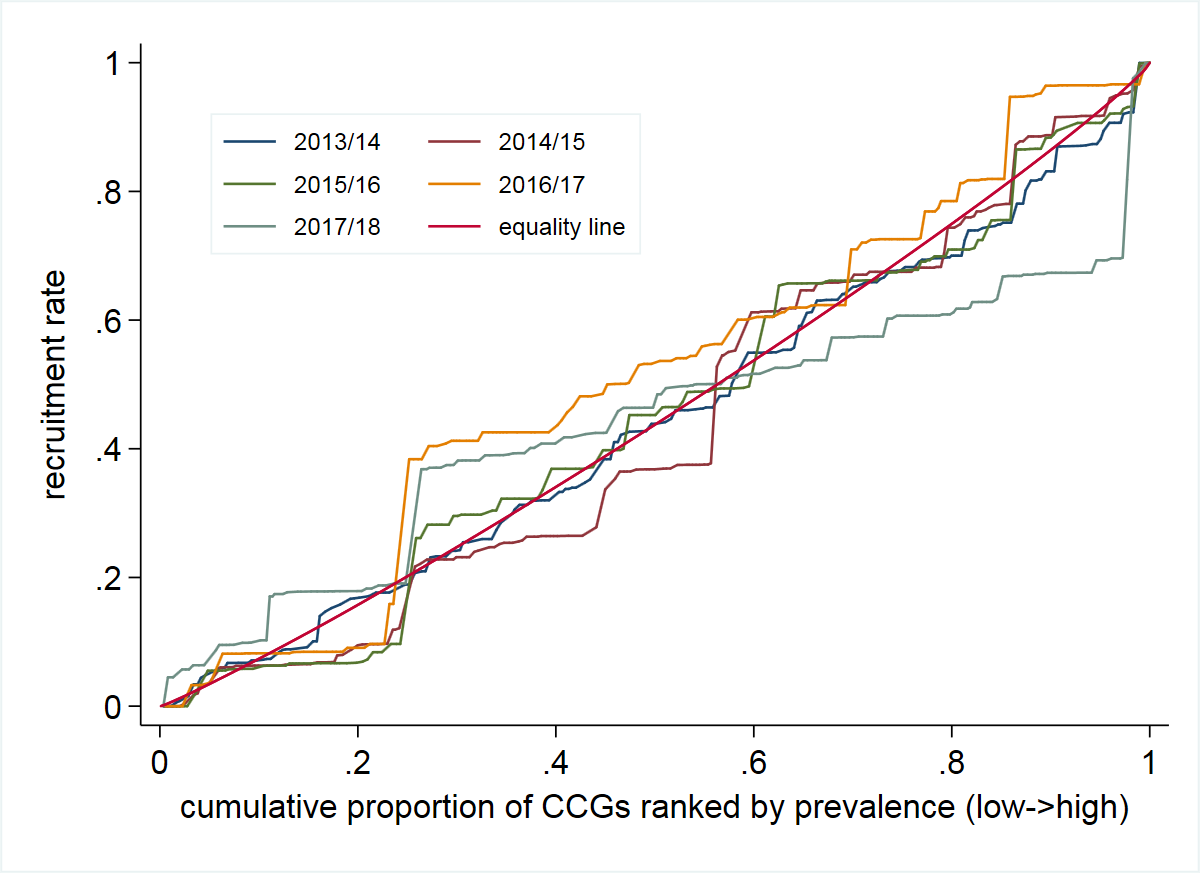
**

| **Appendix Table 4: Univariate regression analysis – Effects of time on redistribution index** | | | | |
| --- | --- | --- | --- | --- |
|  | **CCG**  **Primary care and Trust data** | **CCG**  **Primary care data only** | **LCRN**  **Primary care and Trust data** | **LCRN**  **Primary care data only** |
|  | **All conditions** | | | |
| Time | -0.596 (-0.596, 0.409), <0.001 (0.000) | 0.408 (0.408, 0.409), <0.001 (0.000) | 0.245 (0.245, 0.245), <0.001 (0.000) | 0.997 (0.996, 0.997), <0.001 (0.000) |
| Constant | 46.242 (46.242, 46.243), <0.001 (0.000) | 40.983 (40.982, 40.984), <0.001 (0.000) | 11.767 (11.767, 11.768), <0.001 (0.000) | 16.420 (16.420, 16.421), <0.001 (0.000) |
|  | **Mental Health** | | | |
| Time | 0.036 (0.036, 0.037), <0.001 (0.000) | -4.855 (-4.856, -4.854), <0.001 (0.000) | -2.220 (-2.221, -2.220), <0.001 (0.000) | -5.076 (-5.076, -5.076), <0.001 (0.000) |
| Constant | 57.065 (57.064, 57.066), <0.001 (0.000) | 82.813 (82.811, 82.814), <0.001 (0.000) | 23.725 (23.724, 23.726), <0.001 (0.000) | 59.273 (59.272, 59.274), <0.001 (0.000) |
|  | **Diabetes** | | | |
| Time | 1.078 (1.078, 1.079), <0.001 (0.000) | 4.044 (4.043, 4.044), <0.001 (0.000) | -1.647 (-1.647, -1.646), <0.001 (0.000) | 7.129 (7.129, 7.179), <0.001 (0.000) |
| Constant | 54.281 (54.281, 54.282), <0.001 (0.000) | 56.852 (56.851, 56.853), <0.001 (0.000) | 32.780 (32.799, 32.800), <0.001 (0.000) | 27.018 (27.017, 27.020), <0.001 (0.000) |

95% Confidence Intervals are in brackets, Results are reported as regression coefficients followed by P-values and Standard Errors in parentheses.
